# RNA Structure Design Improves Activity and Specificity of *trans*-Splicing-Triggered Cell Death in a Suicide Gene Therapy Approach

**DOI:** 10.1016/j.omtn.2018.01.006

**Published:** 2018-01-31

**Authors:** Sushmita Poddar, Pei She Loh, Zi Hao Ooi, Farhana Osman, Joachim Eul, Volker Patzel

**Affiliations:** 1 Department of Microbiology & Immunology, Yong Loo Lin School of Medicine, National University of Singapore, Block MD4, Level 5, 5 Science Drive 2, Singapore 117597, Singapore; 2 INEIDFO GmbH, Weserstrasse 23, 12045 Berlin, Germany; 3 Department of Medicine, Division of Infectious Diseases, University of Cambridge, Addenbrooke’s Hospital, Level 5, Hills Road, Cambridge CB2 0QQ, UK

**Keywords:** RNA *trans*-splicing, rational RNA design, suicide gene therapy, hepatocellular carcinoma, HPV-16

## Abstract

Spliceosome-mediated RNA *trans*-splicing enables correction or labeling of pre-mRNA, but therapeutic applications are hampered by issues related to the activity and target specificity of *trans*-splicing RNA (tsRNA). We employed computational RNA structure design to improve both on-target activity and specificity of tsRNA in a herpes simplex virus thymidine kinase/ganciclovir suicide gene therapy approach targeting alpha fetoprotein (AFP), a marker of hepatocellular carcinoma (HCC) or human papillomavirus type 16 (HPV-16) pre-mRNA. While unstructured, mismatched target binding domains significantly improved 3′ exon replacement (3’ER), 5′ exon replacement (5’ER) correlated with the thermodynamic stability of the tsRNA 3′ end. Alternative on-target *trans*-splicing was found to be a prevalent event. The specificity of *trans*-splicing with the intended target splice site was improved 10-fold by designing tsRNA that harbors secondary target binding domains shielding alternative on-target and blinding off-target splicing events. Such rationally designed suicide RNAs efficiently triggered death of HPV-16-transduced or hepatoblastoma-derived human tissue culture cells without evidence for off-target cell killing. Highest cell death activities were observed with novel dual-targeting tsRNAs programmed for *trans*-splicing toward AFP and a second HCC pre-mRNA biomarker. Our observations suggest *trans*-splicing represents a promising approach to suicide gene therapy.

## Graphical Abstract

**Figure:**
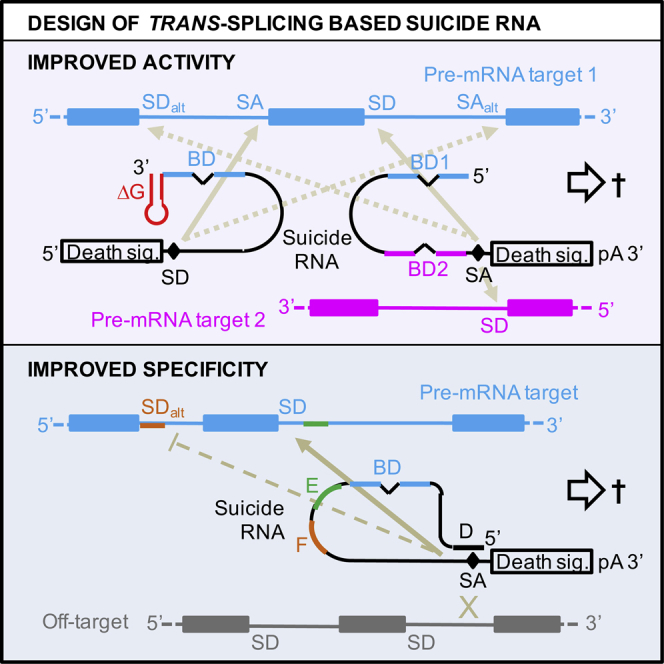

## Introduction

Spliceosome-mediated RNA *trans*-splicing represents a form of alternative splicing in which sequences from distinct pre-mRNAs are joined with the help of the spliceosome to produce chimeric RNAs and proteins. In mammalian cells, *trans*-splicing occurs accidentally or the mechanism is recruited by distinct viruses to generate additional proteins.^1^ Because *trans*-splicing enables labeling and reprogramming of genetic information at the level of the pre-mRNA, researchers have been exploring *trans*-splicing for diagnostic or therapeutic interventions.^2^, ^3^, ^4^, ^5^, ^6^ So far, most efforts have focused on replacement of defective exons for treatment of inherited or acquired genetic disorders.^7^ However, the development of therapies for treatment of human diseases has been limited by low *tran*s-splicing activity and/or specificity and by delivery barriers.^2^, ^4^, ^7^ *trans*-splicing RNA (tsRNA) is generally composed of three functional domains: a splicing domain harboring all recognition motifs required for recruitment of the spliceosome, a domain coding for the polypeptide or protein to be fused to the targeted gene product, and a binding domain (BD).^3^ According to general scientific opinion, advanced BD design represents the key to facilitate applications of the *trans*-splicing technology. State-of-the-art BD design favors long over short BDs to foster *trans*-splicing activity, though it has been speculated that increasing BD length might increase the risk of off-target *trans*-splicing (off-ts).^8^, ^9^, ^10^ In addition, BDs usually bind the pre-mRNA near the targeted splice site (ss) with perfect target complementarity.^11^ Attempts to suppress *cis*-splicing by directing the BD to the *cis*-splice partnering site of the intended target ss were also reported to improve *trans*-splicing efficiencies.^8^, ^12^ Furthermore, directing BDs against introns in which the targeted ss have rather weak *cis*-splice partnering sites was reported to favor splicing in *trans*.^13^, ^14^ The BDs are selected either empirically^15^ or using fluorescence-based screening systems.^16^ However, these experimental approaches are time, cost, and labor intensive, and none of them are capable of considering the whole sequence space of possible BDs for a given target message. Beyond those guidelines, defined rules describing BD design for tsRNA do not exist.

The present study aimed to investigate the role of RNA secondary structures of tsRNA and in particular of the target BDs in RNA *trans*-splicing. We used computational selection, combined with modular RNA design, and derived structure-function relationships that enable a rational design of tsRNA for 5′ or 3′ exon replacement (ER). We split up BD function, selecting primary BDs for efficient target binding and secondary BDs for improved specificity. A main drawback of the *trans*-splicing technology relies on efficiencies not approximating 100%, which makes it difficult to target autosomal dominant genetic disorders. Hence, we explored an advanced tsRNA design toward a herpes simplex virus thymidine kinase (HSVTK)/ganciclovir (GCV) suicide gene therapy approach.^17^, ^18^ As molecular targets, we selected first the alpha fetoprotein (AFP) and three additional markers of hepatocellular carcinoma (HCC)^19^, ^20^ and second human papillomavirus type 16 (HPV-16) pre-mRNA. These targets were selected because the corresponding target organs, the liver, and basal epithelial cells of the skin or mucous membranes are more targetable by non-viral vectors or naked nucleic acids than most other organs.^21^ Using rationally designed tsRNA, we successfully triggered a therapeutically relevant phenotype, i.e., significant and selective cell death, via *trans*-splicing toward endogenously expressed natural precursor mRNA.

## Results

### *De Novo*-Designed tsRNA for 5′ and 3′ ER Triggers Targeted *trans*-Splicing toward an Overexpressed or Endogenous Pre-mRNA Target

**Figure 1:**
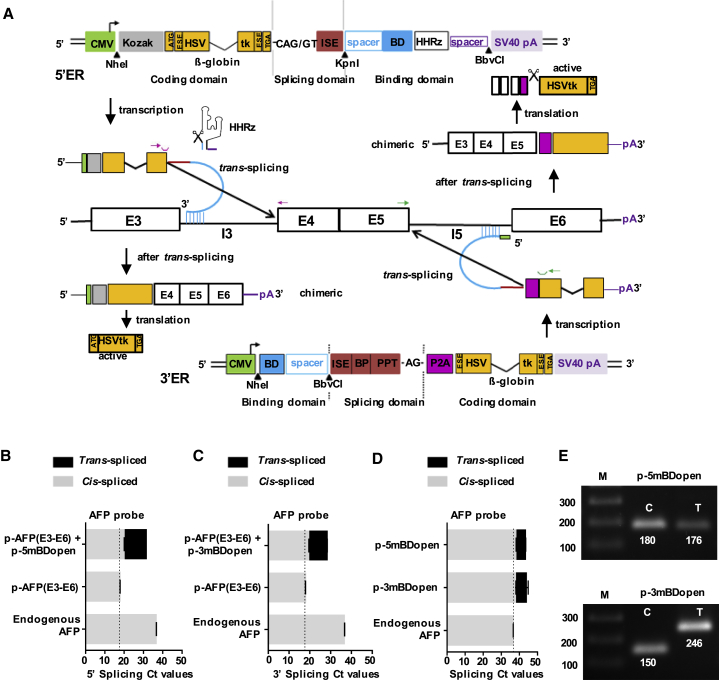
Design and Functional Investigation of *trans*-Splicing-Based Suicide RNA (A) Design of tsRNA for 5′ and 3′ exon replacement (ER) and structure of an AFP mini-gene. The AFP mini-gene encompasses AFP exons 3–6 (E3–E6), including introns 3 and 5 (I3 and I5) but lacking intron 4. The tsRNAs comprise three functional domains. First, computationally selected 50 nt binding domains (BDs) harboring central 2 nt target mismatches, which are fused to the other domains via spacers (blue); the BDs of the 5′ or 3′ ER tsRNAs bind to AFP I3 or I5. Second, splicing domains composed of an intronic splice enhancer (ISE) and a consensus splice donor (SD) site (CAG/GT) for 5′ ER or an ISE, a consensus YNYURAC branchpoint (BP) sequence, an extensive polypyrimidine tract (PPT), and a consensus splice acceptor (SA) site (AG/G) for 3′ ER (red). Third, HSVtk coding domain occuring with or without a start codon for 5′ or 3′ ER, including a novel exonic splice enhancer (ESE), as well as a β-globin mini-intron (orange). The tsRNA for 5′ ER additionally harbors a tertiary structure-stabilized hammerhead ribozyme (HHRz) downstream of the BDs; the tsRNA for 3′ ER is featured with a P2A proteolytic cleavage site upstream of the HSVtk (magenta). For detection of *trans*-splicing, positioning of primers (arrows) and probes (open troughs) for TaqMan probe-based real-time RT-PCR detection is indicated in magenta (5′ ER) and green (3′ ER). (B–D) Real-time RT-PCR quantification of *trans*- and *cis*-spliced RNA isolated from transfected HepG2 cells. Indicated are raw C_t_ values, mean ± SEM (n = 3). (B) Detection of *trans*-splicing toward the overexpressed and endogenous AFP message in cells transfected with the parental 5′ ER construct 5-mBDopen and/or the AFP mini-gene p-AFP (E3–E6). (C) Detection of *trans*-splicing toward the overexpressed and endogenous AFP message in cells transfected with the parental 3′ ER construct 3-mBDopen and/or the AFP mini-gene p-AFP (E3–E6). (D) Detection of *trans*-splicing toward the endogenous AFP message in cells transfected with the 5′ or 3′ ER construct. (E) RT-PCR (35 + 35 cycles) detection of endogenous AFP mRNA after *cis*-splicing (C) or *trans*-splicing (T) via 5′ ER (upper panel) or 3′ ER (lower panel) on 1% agarose gels. See also Figure S1 and Table S1.

We designed parental tsRNA molecules for maximum 5′ or 3′ ER activity comprising the following molecular features (Figure 1A). First, unstructured BDs 50 nt in length binding to introns 3 or 5 of the AFP pre-mRNA were computationally selected out of the complete antisense RNA (asRNA) structure space that can be directed against all 14 introns of the AFP target message; in addition, we selected a spacer that preserves selected BD structures in the context of the complete tsRNA molecule. We implemented central 2 nt target mismatches into the selected BDs, generating mismatched BDs (mBDs). This was to prevent formation of long nuclear double-stranded RNA, which could trigger antisense effects, including adenosine-to-inosine (A-to-I) editing by adenosine deaminases acting on RNA (ADARs),^22^ impairing the *trans*-splice strategy. Second, a splicing domain was composed of all consensus splice signals to compete with cellular *cis*-splicing. Third, a coding domain was composed of an optimized HSVtk gene harboring a novel exonic splice enhancer (ESE), as well as the β-globin mini-intron (Figures S1A and S1B). In addition, we furnished the tsRNA for 5′ ER with a tertiary structure-stabilized hammerhead ribozyme (HHRz)^23^ positioned downstream of the BD. This HHRz could then crop itself, together with the simian virus 40 (SV40) poly(A) site, to trigger nuclear RNA retention and to avoid *trans*-splicing-independent HSVTK expression. A similar strategy using a first-generation HHRz had no effect on *trans*-splicing.^11^ Here, formation of the active ribozyme structure was additionally fostered by inserting a spacer between the ribozyme and the poly(A) site. The tsRNA for 3′ ER was featured with the 2A peptide derived from porcine teschovirus-1 (P2A) proteolytic cleavage site positioned downstream of the splice A site to trigger proteolytic release of the HSVTK from the chimeric fusion protein, which results from the 3′ ER *trans*-splice reaction. Optimized parental constructs for 3′ and 5′ ER were termed p-3mBDopen and p-5mBDopen, respectively. As controls, we designed two ss mutants (Δss1 and Δss2) each, a partly inactive HSVtk mutant (ΔHSV), and for 5′ ER, a control harboring an inactive HHRz cleavage site (ΔHH) (Table S1). Based on the binding sites of computationally selected BDs, we designed an overexpressing AFP mini-gene (Figure 1A). Endogenous AFP mRNA and protein expression were detected in HEK293T cells and in the liver cancer cell lines HepG2, PLC/PRF5, SNU495, and CL48 (Figure S1C). HepG2 cells were cotransfected with the *trans*-splicing vector, and the AFP mini-gene and levels of *trans*-spliced and *cis*-spliced AFP RNA were quantified by real-time RT-PCR using an AFP-specific TaqMan probe. *trans*-splicing was additionally detected using an HSVtk-specific probe (Figure S1D). *cis*-splicing was found to be more prevalent than *trans*-splicing, and the ss mutants revealed reduced *trans*-splice activity. Competition between *cis*- and *tran*s-splicing was indicated by a reduction of *cis*-splice levels in the presence of *tran*s-splicing (Figures 1B–1D). The accuracy of *trans*- or *cis*-splicing toward or within both the overexpressed and the endogenous AFP transcript was confirmed by cDNA sequencing (Figure 1E; Figure S1E). *trans*-splicing-triggered HSVTK protein expression was monitored by western blot analyses and found to correlate well with the levels of *trans*-splice RNA (Figures S1F–S1I). Although 3′ ER triggered the formation of a 42 kDa HSVTK isoform, 5′ ER and the HSVTK positive control (HSVTK^+^) lead to the expression of 42 and 44 kDa isoforms. No chimeric AFP-HSVTK fusion proteins were detectable with the 3′ ER constructs, pointing toward efficient P2A proteolytic cleavage. In the case of 5′ ER, the ss mutants triggered clearly reduced levels of HSVTK expression compared with the parental construct, indicating most HSVTK protein originated from *trans*-splicing, not from leaky splicing-independent expression.

### Design of BD Sequence and Structure Substantially Improves 3′ ER, but Not 5′ ER

**Figure 2:**
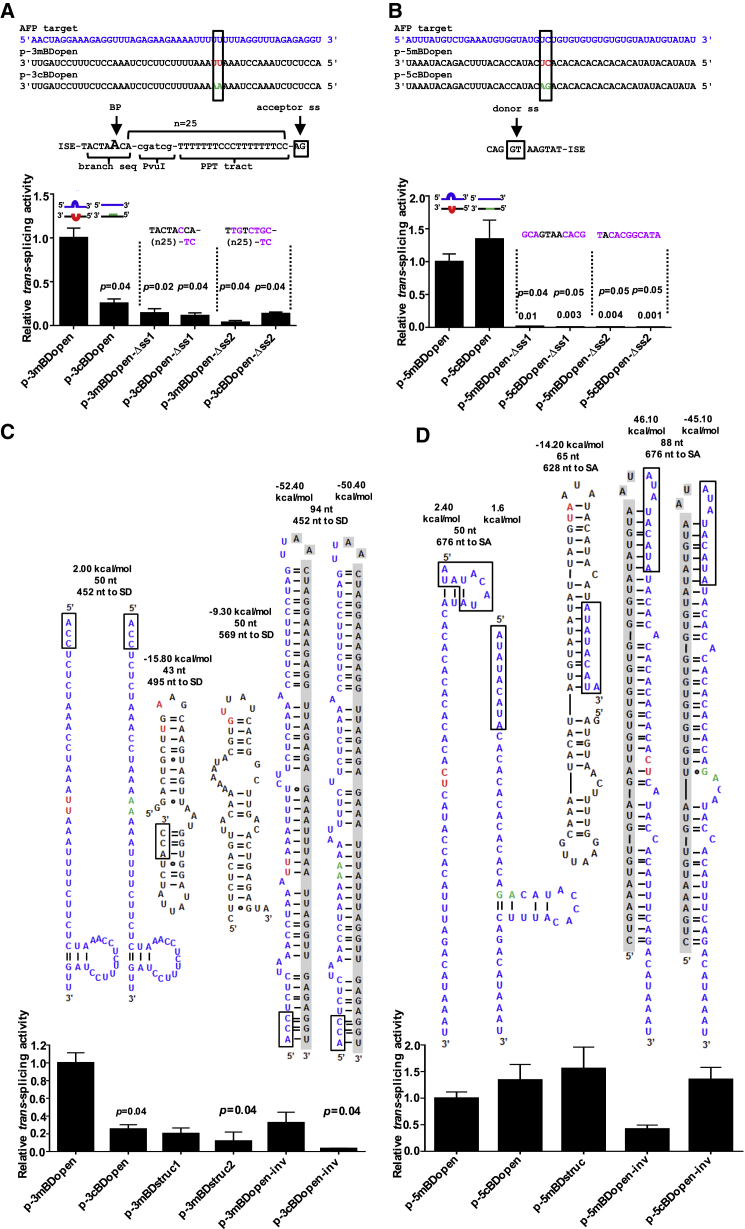
Binding Domain Sequence and Structure Design Improves 3′ ER, but Not 5′ ER, Activity Computationally selected unstructured parental BDs for 3′ ER (A) or 5′ ER (B) of 50 nt in length were tested either as conventionally designed sequences with perfect complementary to the AFP target (3cBDopen and 5cBDopen) or harbored 2 nt target mismatches (3mBDopen and 5mBDopen) (upper panel). For each construct, two controls with mutated splice sites (−Δss1 and −Δss2) were generated, encompassing branchpoint and splice acceptor mutations for 3′ ER or splice donor mutations for 5′ ER. Relative *trans*-splice activities were detected by real-time RT-PCR quantification of *trans*-spliced RNA isolated from transfected HepG2 cells (lower panel). Blue, target sequence; red, target mismatches in BDs; green, corresponding complementary positions; magenta, splice site mutations. PPT, polypyrimidine tract; BP, branchpoint. RNA secondary structures as predicted by mfold of computationally selected BD structures for 3′ ER (C) or 5′ ER (D) of 43 to 94 nt in length (upper panels). Relative *trans*-splice activities were detected by real-time RT-PCR quantification of *trans*-spliced RNA isolated from transfected HepG2 cells (lower panels). Gibbs free energy (ΔG) of secondary structure formation and distances of the BD binding site to the nearest target splice donor (SD) or splice acceptor (SA) sites are indicated. Blue, sequence of unstructured parental BDs; red, target mismatches; green, corresponding complementary positions; boxes, overlapping BD segments; gray shading, inverted internal repeats binding the parental BD sequences. *trans*-splicing activities were monitored 24 hr post-transfection. Mean ± SEM (n = 3). Significance was tested using one-way ANOVA with Tukey post hoc test. Relative RNA levels were calculated in terms of fold change (2^−ΔΔCt^), where ΔC_t_ = C_t *trans*-spliced AFP_ − C_t β-Actin_. See also Figures S2–S5.

The tsRNA BDs can be considered asRNAs that are fused to the splicing and coding domains. Hence, it is reasonable to assume that the same rules govern the design of effective asRNA and BDs for tsRNA; i.e., unstructured, flexible, and short BDs should be favored over structured, rigid domains.^24^ We investigated the role of BD RNA secondary structure in RNA *trans*-splicing. Using our software tool foldanalyze,^24^ we identified the least structured BDs of 50 nt in length that can be targeted against the AFP pre-mRNA: 3mBDopen or 5mBDopen for 3′ or 5′ ER binding to intron 5 or intron 3, respectively (Figure 2). As controls, we identified highly structured domains of comparable lengths (3mBDstruc1, 3mBDstruc2, and 5mBDstruc), some of which overlapped with the unstructured domains, as well as two mBDs (3mBDopen-inv and 5mDBopen-inv), by employing internal inverted repeats fully encompassing the favorable BDs (3mBDopen and 5mBDopen), turning them into structured, unfavorable BDs but maintaining the binding site and distance to the target ss (Figures S2 and S3). Selected BDs were fused to the tsRNA via spacer sequences to preserve the selected BD structures (Figure S4). To suppress RNA editing, all BDs harbored central 2 nt target mismatches. To investigate the effect triggered by these mismatches, we additionally generated fully complementary BD (cBD) analogs 3cBDopen, 5cBDopen, 3cBDopen-inv, and 5cBDopen-inv (Figures 2A and 2B). All cBDs and mBDs were cloned into the parental *trans*-splicing vectors. RNA was isolated from HepG2 cells cotransfected with the *trans*-splicing plasmids and the AFP mini-gene vector. In the case of 3′ ER, the tsRNA harboring 3mBDopen triggered 4-fold (p = 0.04), 3-fold, or 30-fold (p = 0.04) stronger *trans*-splicing compared with its analogs harboring 3cBDopen, 3mBDopen-inv, or 3cBDopen-inv, respectively (Figure 2C). In addition, 3mBDopen was 4- or 6-fold (p = 0.04) more potent than 3cBDstruc1 or 3cBDstruc2. These data indicate mBDs are more effective than cBDs and unstructured BDs are more efficient than structured ones. Conversely, the degree of RNA secondary structure formation and target complementarity within the BDs did not affect 5′ ER activities (Figure 2D). ss mutations impaired 3′ and 5′ ER activities of all constructs (Figures 2A and 2B; Figure S5).

### 5′ ER Correlates with the Thermodynamic Stability of the tsRNA 3′ End

**Figure 3:**
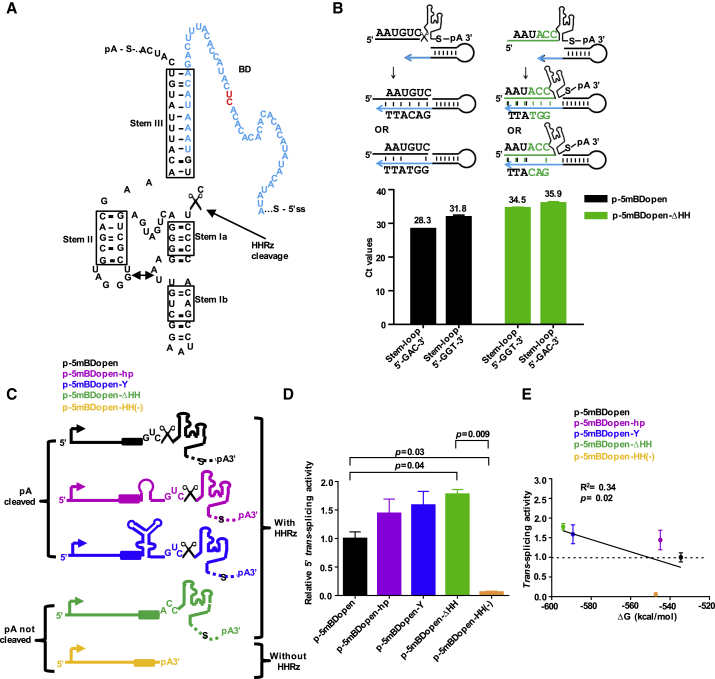
Activities of 5′ ER Correlate with the Thermodynamic Stability of RNA 3′ Ends, which Can Be Processed by a HHRz (A) RNA secondary structure as predicted by mfold of a tertiary structure-stabilized HHRz designed for *cis*-cleavage of tsRNA 3′ ends. The HHRz is attached downstream of the 3′ end of the target BD (blue), with indicated target mismatches (red). The cleavage site following the GUC cleavage motif is indicated by a scissor icon. Stems are highlighted in boxes; the double-headed arrow indicates tertiary structure interactions between the stem II hairpin loop and the stem I bulge. (B) Investigation of HHRz cleavage toward the active GUC or the inactive ACC cleavage motif using stem-loop primer-based real-time RT-PCR quantification of processed tsRNA 3′ ends. Blue arrow, 3′ end binding site of hairpin primer; green, mutated HHRz cleavage motif. Mean values of raw C_t_ values ± SEM (n = 3). (C) Schematic representation of tsRNAs for 5′ ER harboring different 3′ end stabilizing RNA secondary structures downstream of the BD and/or an active or inactive HHRz. (D) 5′ ER activities of tsRNA with different thermodynamic 3′ end stabilities relative to the parental construct p-5mBDopen harboring the active HHRz cleavage site (black). Mean ± SEM (n = 3). Significance was tested using one-way ANOVA with Tukey post hoc test. Relative RNA levels were calculated in terms of fold change (2^−ΔΔCt^), where ΔC_t_ = C_t *trans*-spliced AFP_ − C_t β-Actin_. (E) Correlation between relative *trans*-splicing activities and the Gibbs free energy (ΔG) of RNA secondary structure formation of the 5′ ER tsRNAs with different 3′ ends. Linear regression analysis was done using Prism 6 software. R^2^ = correlation coefficient, mean ± SD (n = 3). See also Figures S1D and S6.

Construct p-5mBDopen-ΔHH harboring the inactive HHRz cleavage motif triggered stronger *trans*-splice activities compared with its cleavable analog p-5mBDopen (Figure S1D, upper panel). That was surprising, because the uncleaved RNA comprises a poly(A) tail and is expected to be exported to the cytoplasm, thereby lowering its nuclear concentration and the rate of *trans*-splicing. Hence, we investigated cleavage of the active and inactive HHRz motifs by quantifying the cleaved RNA 3′ end using stem-loop primer-based real-time RT-PCR^25^ (Figures 3A and 3B). This method was proven to efficiently discriminate between perfect primer-matching and extended RNA 3′ ends. The observed 6.2 cycle difference indicated a ribozyme cleavage rate above 90% (active motif), yielding tsRNA with an unstructured 3′ terminal BD (Figure 3B). Unstructured ends of asRNA were reported to be associated with both fast target binding and degradation.^24^ To stabilize the 3′ end of the tsRNA for 5′ ER after ribozyme cleavage, we designed constructs harboring a hairpin-loop (p-5mBDopen-hp) or Y-shaped (p-5mBDopen-Y) structure downstream of the BD but upstream of the HHRz cleavage site. In another control, the ribozyme and the spacer were removed so that the BD was directly followed by the poly(A) site (p-5mBDopen-HH(−)) (Figure 3C). RNA secondary structure predictions using mfold^26^ and RNAfold^27^ supported correct folding of the stabilizing domains, the HHRz, and openness of the BDs after ribozyme cleavage (Figure S6). Experimental investigation of all 5′ ER constructs revealed a positive correlation between the thermodynamic stabilities of the RNA 3′ ends, i.e., the Gibbs free energy (ΔG) of secondary structure formation, and the *trans*-splicing activities (Figures 3D and 3E). Strongest *trans*-splicing was observed for the construct with the inactive HHRz cleavage site harboring a stable secondary structure formed by the spacer and the SV40 poly(A) site, followed by the constructs with the terminal Y-shaped and hairpin-loop structures and the parental construct with the 3′ terminal BD. By far the lowest *trans*-splice activity was measured for the construct comprising the poly(A) site but lacking a stable structure at the 3′ end.

### Alternative On-Target *trans*-Splicing Is a Prevalent Event that Competes with Targeted *trans*-Splicing

**Figure 4:**
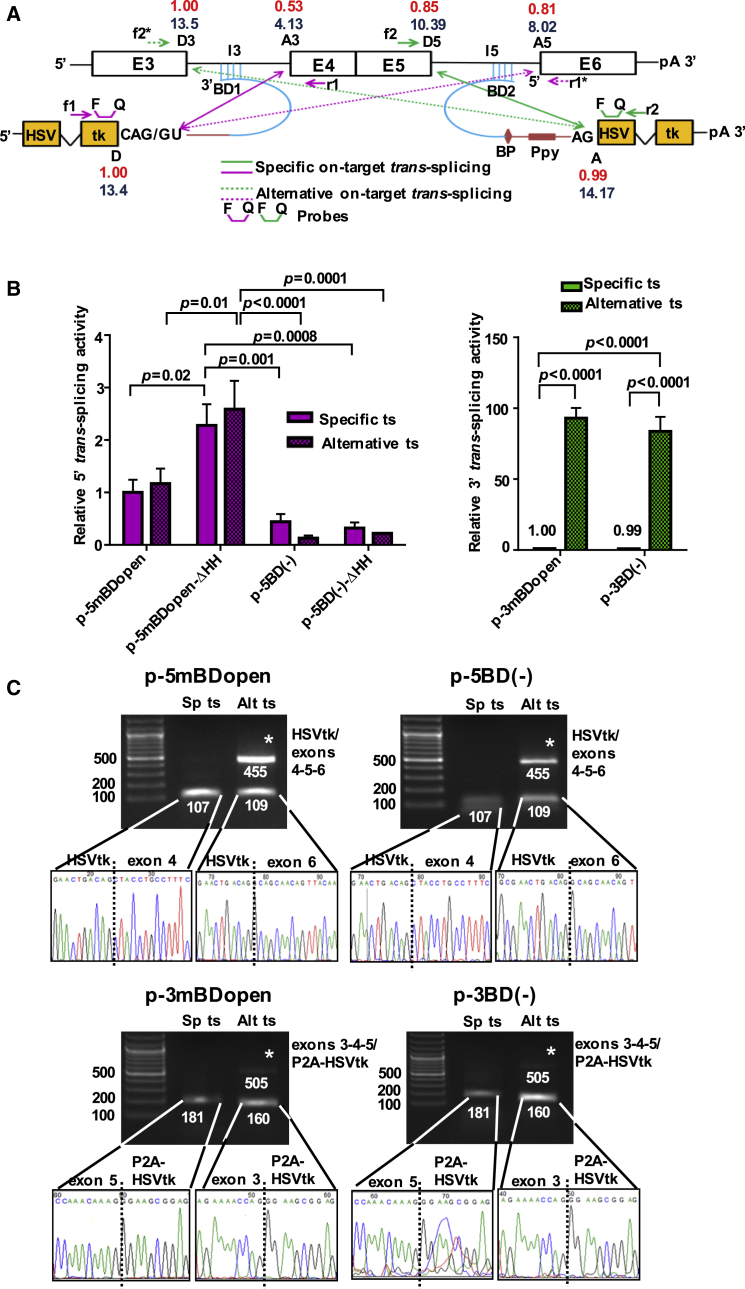
Alternative On-Target *trans*-Splicing Is a Prevalent Event (A) Schematic drawing depicting specific on-target *trans*-splicing (sp-on-ts) and alternative on-target *trans*-splicing (alt-on-ts) between the parental tsRNAs for 5′ and 3′ ER and the AFP mini-gene transcript. Solid arrows indicate sp-on-ts, and dashed arrows show alt-on-ts events. Numbers in red or blue denote the strengths of the donor (D) and acceptor (A) splice sites as calculated using SSP or ASSP algorithms, respectively. Positioning of primers and probes for TaqMan probe-based real-time RT-PCR detection is indicated. D and A indicate splice donor and acceptor sites of the tsRNA; D3 and D5 or A3 and A5 indicate splice D or A sites of the AFP mini-gene RNA. PCR forward primers are f1, f2*, and f2; reverse primers are r1, r1*, and r2. For further details, see the legend of Figure 1A. (B) Relative sp-on-ts and alt-on-ts activities triggered by different tsRNAs for 5′ ER (left) and 3′ ER (right) measured using real-time RT-PCR. Means ± SEM (n = 3). Significance was tested using two-way ANOVA with Bonferroni post hoc test. (C) RT-PCR (30 + 30 cycles) detection of chimeric RNA resulting from sp-on-ts and/or alt-on-ts between the overexpressed AFP mini-gene transcript and the 5′ or 3′ ER constructs via 1% agarose gel electrophoresis (upper panel) or DNA sequencing (lower panel). *Upper bands resulting from a combination of specific on-target *trans*-splicing and *cis*-splicing of the respective second intron detected with primers f1 and r1* (5′ ER) or f2* and r2 (3′ ER). See also Table S2 and Figure S14.

The target ss that is closest to the BD binding site is assumed to be recruited for *trans*-splicing. However, we reported that in the case of viral *trans*-splicing, it is the strength of the available splice donor (D) and splice acceptor (A) sites within the target that rules the *trans*-splice process.^13^ The use of strong SD and SA sites in tsRNA can also initiate spliceosome E-complex formation and thus *trans*-splicing in the absence of a BD,^11^ although target-specific BDs significantly enhance *trans*-splicing.^28^, ^29^ We compared the efficiencies of specific on-target *trans*-splicing (sp-on-ts) versus alternative on-target *trans*-splicing (alt-on-ts) between the AFP mini-gene pre-mRNA and BD^+^, as well as BD(–), tsRNAs using real-time RT-PCR (Figure 4). Possible *trans*-splice reactions, predicted ss strengths, and the positioning of primers and probes are depicted in Figure 4A. We found alt-on-ts to represent a highly prevalent event. In 3′ ER, alt-on-ts was up to 90-fold more frequent than sp-on-ts; in 5′ ER, both reactions were equally abundant (Figure 4B). Specific on-target 3′ or 5′ ER triggers the formation of exon-5/HSVtk or HSVtk/exon-4 chimeric RNA, respectively. Alternative on-target 3′ or 5′ ER leads to the formation of exon-3/HSVtk or HSVtk/exon-6 chimera. All chimeric RNAs were detected using PCR, and exon junctions were confirmed by sequencing (Figure 4C). Primers and probes used for alt-on-ts detection additionally amplified a second, longer chimeric RNA of the type exons-3-4-5/HSVtk or HSVtk/exons-4-5-6, which results from the sp-on-ts reaction. Deletion of the AFP-specific BD reduced 5′ ER activities 2- to 3-fold but did not impair the 3′ ER activities (Figure 4B), possibly due to accidental complementarities between the BD(–) tsRNA and the target (Table S2). We investigated *tran*s-splicing of our BD(–) RNAs toward highly abundant cellular pre-mRNAs, including GAPDH and β-actin, but couldn’t detect any off-ts.

### Secondary BDs Enhance Targeted *trans*-Splicing and Suppress alt-on-ts, Increasing the Specificity of 3′ ER

**Figure 5:**
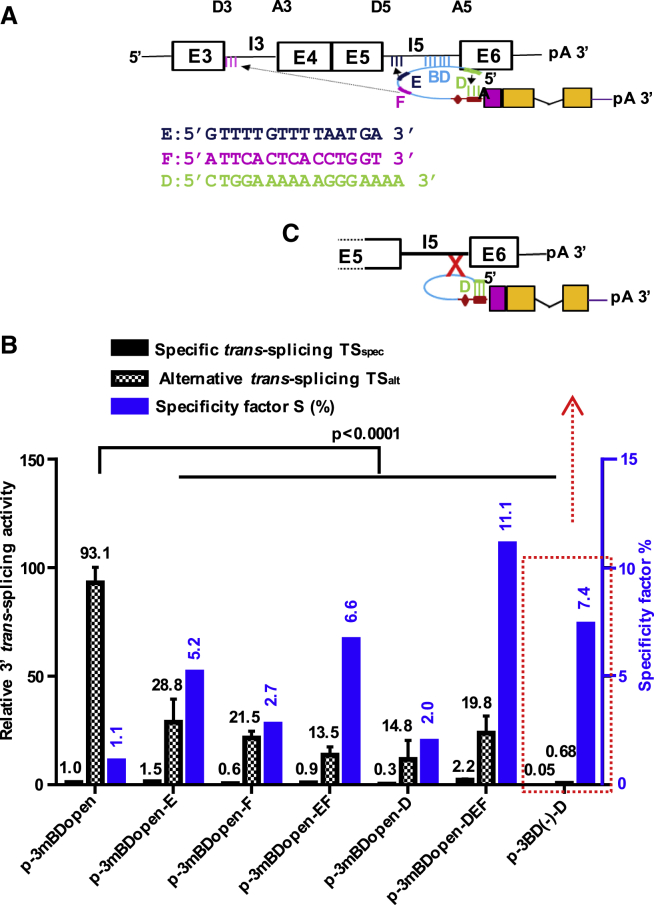
Improvement of *trans*-Splicing Specificity by Implementation of Secondary Binding Domains (A) Schematic drawing showing design of tsRNA for 3′ ER harboring secondary, as well as the respective, BD sequences and binding sites. Secondary BDs D, E, and F are 15 to 17 nt in length and fully complementary to the target. Blue, computationally selected unstructured BD 3mBDopen designed for fast target binding; green, BD D binding to PPT tract of the tsRNA; dark blue, BD E binding close to splice donor D5 of I5; magenta, BD F binding to splice donor D3 of I3. (B) Relative activities of specific (*TS*_spec_, solid black bars) and alternative (*TS*_alt_, checkered bars) on-target *trans*-splicing detected by real-time RT-PCR isolated from HepG2 cells cotransfected with the AFP mini-gene and different *trans*-splicing constructs both referring to the left y axis and a specificity factor (*S*, blue bars) referring to the right y axis. The specificity factor was calculated using the equation *S* = *TS*_spec_/*TS*_alt_ × 100%. Mean ± SEM (n = 3). Significance was tested using two-way ANOVA with Tukey post hoc test. (C) Schematic drawing of construct p-3BD^−^-D, lacking a target BD but including BD D. See also Figure S7.

To suppress alt-on-ts and to improve *trans*-splicing specificity, we designed tsRNAs for 3′ ER harboring multiple target and/or self-BDs (Figures 5A and 5B). Supplementary to 3mBDopen, which has been optimized for fast target binding, we implemented several secondary BDs: secondary BD E brings the intended *trans*-ss donor (D)5 and acceptor (A) closer to each other, secondary BD F binds and functionally blocks the alternative ss D3, and self-BD D was positioned directly upstream of 3mBDopen to shield the PPT of ss acceptor A of the tsRNA in the absence of target binding (Figure 5C). A safety stem approach reported earlier could suppress off-ts but also decreased *trans*-splice activity.^30^ In addition, we generated constructs with multiple BDs harboring BDs E and F or BDs D, E, and F complementary to 3mBDopen. All secondary BDs increased the specificity of *trans*-splicing, as reflected by an increase in the specificity factor (Figure 5B). While BD F and the more pronounced BD D suppressed both sp-on-ts and alt-on-ts, BD E and the combination of BDs E and F enhanced sp-on-ts but suppressed alt-on-ts. Most successful was the combination of all three secondary BDs, which enhanced sp-on-ts about 2-fold and reduced alt-on-ts about 5-fold, thus exhibiting a 10-fold higher specificity of *trans*-splicing compared with the parental construct. In the BD(–) construct, internal BD D suppressed *trans*-splicing toward the strong splice donor D3 or the moderately strong donor D5 20-fold or 130-fold, presuming *trans*-splicing to any other cellular off-targets was suppressed to a similar extent. A comparable reduction of *trans*-splicing was observed in BD(–) constructs harboring mutated ss (Figure S7).

### *trans*-Splicing toward Overexpressed or Endogenous AFP Pre-mRNA Triggers Death in a Human Liver Carcinoma Cell Line

**Figure 6:**
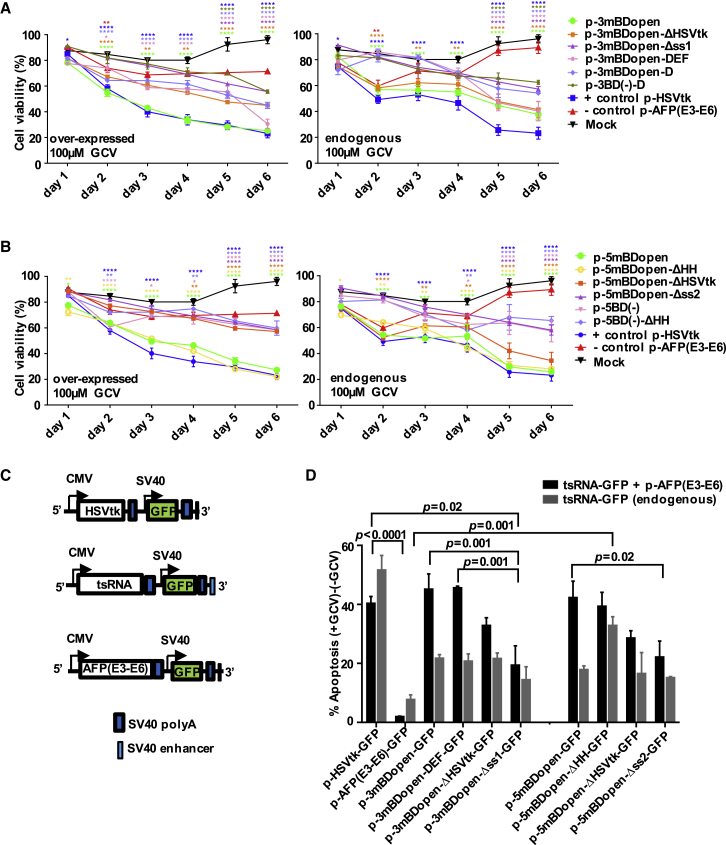
Death of AFP-Expressing HepG2 Cells Triggered by *trans*-Splicing-Based Suicide Vectors AlamarBlue cell viability assay with (A) 3′ ER or (B) 5′ ER constructs using cells with overexpressed (left panels) or endogenous (right panels) AFP levels and 100 μM GCV over a period of 6 days. BD-optimized constructs strongly reduced cell viability. (A and B) Mean ± SEM (n = 3). Significance was tested using two-way ANOVA with Bonferroni post hoc test compared to mock. (C) Design of vectors harboring the *egfp* gene. (D) Apoptosis triggered by 3′ and 5′ ER constructs 48 hr post-transfection using 100 μM GCV in cells with overexpressed (black bars) or endogenous (gray bars) AFP levels as detected using the Annexin V/PI apoptosis assay. Mean ± SEM (n = 3). Significance was tested using two-way ANOVA with Tukey post hoc test. Besides the indicated significances, effects triggered by all samples except p-3mBDopen-Δss1-GFP were significant (p = 0.0008 to < 0.0001) compared with the negative control p-AFP(E3–E6)-GFP in AFP-overexpressing cells. Likewise, effects triggered by all samples were significant (p = 0.04 to < 0.0001) compared with the positive control p-HSVtk-GFP in cells expressing endogenous AFP levels. *p < 0.05, **p < 0.01, ***p < 0.001, and ****p < 0.0001. See also Figures S8–S11.

We chose the HSVtk/GCV gene-directed suicide prodrug therapy approach.^18^ We investigated death of HepG2 cells triggered by 5′ or 3′ ER toward overexpressed and endogenous AFP pre-mRNA in the presence or absence of GCV using three assays. The first was the alamarBlue cell viability assay. Highest levels of cell death, i.e., up to 80% at 100 μM GCV (Figures 6A and 6B) or 60% to 70% at 10 μM GCV (Figure S8), reaching the levels of the positive control, were triggered by *trans*-splicing of the parental constructs toward the overexpressed AFP message at day 6 of the treatment. Likewise, high levels of cell death were observed for the 3′ ER construct containing all four BDs and for the 5′ ER construct with the mutated HHRz cleavage motif. 3′ ER with the endogenous AFP RNA was slightly less efficient. Smaller effects were triggered by the ΔHSV, the ss mutants, and RNAs lacking an AFP-specific BD. No cytotoxicity was triggered by the AFP mini-gene or the *tran*s-splicing constructs in the absence of GCV. Second, to monitor specific modes of cell death, we employed the Annexin V/propidium iodide (PI) apoptosis assay. In initial assays, pEGFP was added to the transfection reactions, but a substantial fraction (∼30%) of apoptotic cells appeared to be EGFP-negative, indicating sub-optimal cotransfection efficiencies (Figure S9). To enable direct gating of successfully transfected cells, *trans*-splicing vectors, the AFP mini-gene vector, and the HSVtk vector were furnished with the *egfp* gene (Figures 6C and 6D; Figure S10). 3′ ER constructs with optimized or multiple BDs and 5′ ER constructs harboring the HHRz (active or inactive cleavage motif) triggered apoptosis in more than 40% or 20% of the AFP-overexpressing cells or cells expressing endogenous AFP, respectively. An exception was the 5′ ER construct with the mutated HHRz cleavage site, which triggered comparable high levels of apoptosis with endogenous or overexpressed AFP. The 5′ ER construct with the mutated HHRz cleavage site 5′ mBDopen-ΔHH represents a fully intact mRNA harboring a 5′ cap and a 3′ poly(A) tail even without undergoing *trans*-splicing. Hence, the high cell death activity triggered by this tsRNA might be due to *trans*-splicing-independent nuclear export and HSVtk translation. Alternatively, the high activity might result from high endogenous RNA stability and corresponding high rates of *trans*-splicing, which were measured for this RNA (Figure 3D). The HSVTK/GCV system was reported to predominantly trigger apoptotic cell death.^31^ To confirm that, we performed the third comet assay, which allows visualization of partially degraded DNA using single-cell gel electrophoresis. The results are in accordance with those obtained in the alamarBlue and Annexin V/PI assays: most DNA breaks were triggered by the multiple BD or parental 3′ ER RNAs or by the 5′ ER RNAs harboring the wild-type or mutated HHRz cleavage site (Figure S11).

### tsRNA Simultaneously Targeting Two Endogenous Liver Cancer Markers Triggered Enhanced Cell Death at 10-Fold Lower GCV Concentration

**Figure 7:**
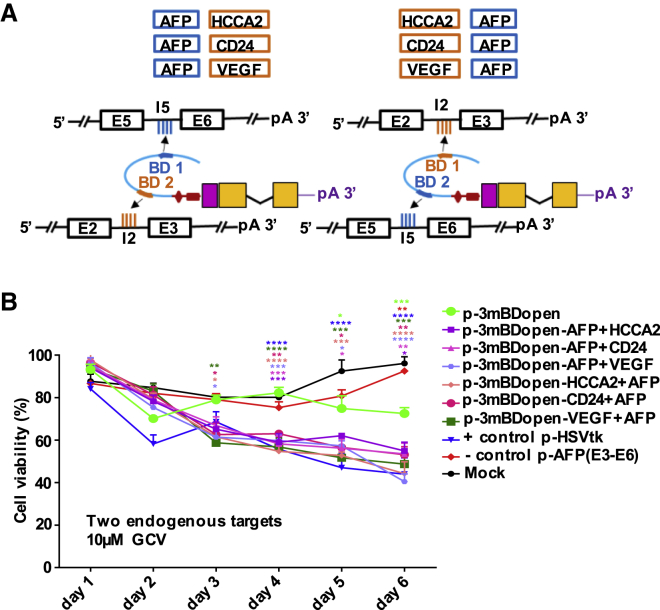
Dual-Targeting *trans*-Splicing RNA Triggers Enhanced Cell Death at 10-Fold Lower GCV Concentration (A) Design of dual-targeting tsRNA for 3′ ER furnished each with two BDs (BD1 and BD2) specific for AFP and one of three alternative HCC biomarkers (HCCA2, CD24, or VEGF) (lower panel). All six combinations or orders of BDs were considered (upper panel). AFP BD 3mBDopen binds to AFP I5, and the additional BDs target I2 of the respective second target genes. Additional BDs were computationally selected to be unstructured binding target introns encompassing weak *cis*-splice partnering sites. (B) alamarBlue cell viability assay comparing the levels of death triggered by dual-targeting and the parental AFP-only targeting constructs in cells expressing endogenous AFP levels after 10 μM GCV treatment. Mean ± SEM (n = 3). Significance was tested using two-way ANOVA with Bonferroni post hoc test compared to mock. *p < 0.05, **p < 0.01, ***p < 0.001, and ****p < 0.0001. See also Figure S12.

The carcinogenesis of HCC is a complex, multifactorial, multistep process, and a single biomarker cannot accurately indicate the disease and its stages.^32^ To increase HCC specificity and sensitivity, we investigated bispecific tsRNAs targeting two HCC biomarkers simultaneously. Of the multiple reported HCC biomarkers,^19^, ^20^ we quantified 12 pre-mRNAs and mRNAs in 10 cell lines or cells (Figure S12A). We selected HCCA2/YY1AP1, CD24, and VEGF as secondary targets and designed dual-targeting tsRNAs, considering all six combinations or orders of the AFP-specific BDs with a BD specific for a secondary target. BDs were selected to be unstructured and to harbor target mismatches and were linked via spacers preserving the selected structures. We investigated death of HepG2 cells targeting the endogenous pre-mRNA targets using the alamarBlue cell viability assay (Figures 7A and 7B). At 10 μM GCV, all dual-targeting constructs triggered significantly higher levels of cell death compared with the construct targeting AFP only (Figure 7B); however, at 100 μM GCV, single- and dual-targeting constructs showed comparable effects (Figure S12B). This is reasonable, because regardless of the GCV concentration, the same number of cells receives the *trans*-splicing vectors. Whereas only the most active tsRNAs can trigger enough death signal at the lower GCV concentration, even less active tsRNAs might reach that threshold at a higher drug concentration.

### HPV-16-Targeting Suicide RNA Specifically Killed HPV-16-Transformed Tissue Culture Cells

**Figure 8:**
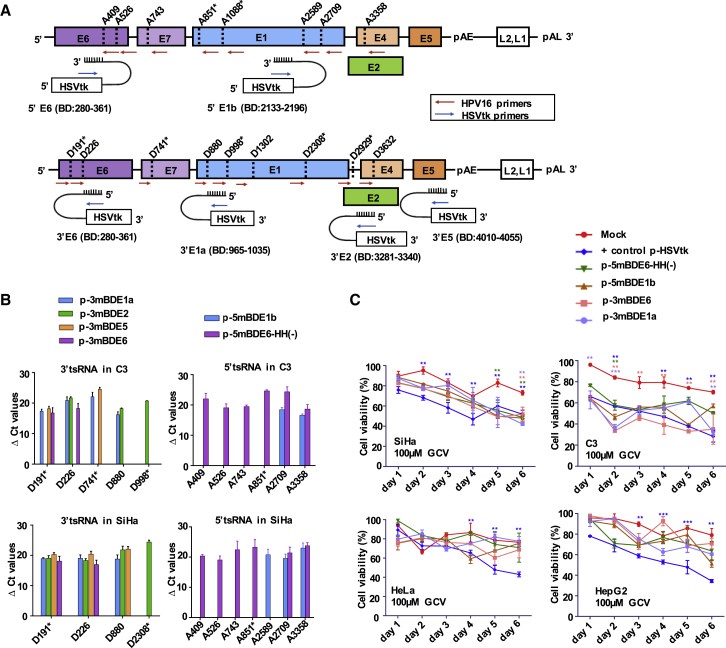
Targeting of HPV-16^+^ Cells with *trans*-Splicing-Based Suicide Vectors (A) Schematic map of the HPV-16 genome containing six early (E) and two late (L) genes with positioning of reported splice acceptor (A) or donor (D) sites, as well as splice sites predicted (*) using ASSP software. We designed two tsRNAs for 5′ ER (5mBDE6-HH(−) and 5mBDE1b) (upper panel) and four tsRNAs for 3′ ER (3mBDE6, 3mBDE1a, 3mBDE2, and 3mBDE5) (lower level). Red arrows indicate positioning of reverse (5′ ER) and forward (3′ ER) primers binding to HPV-16 transcripts; blue arrows indicate positioning of forward or reverse primers in the HSVtk region of the *trans*-splicing constructs. (B) Real-time RT-PCR quantification of *trans*-splicing between the 3′ ER (left panel) and the 5′ ER (right panel) constructs and endogenous HPV-16 transcripts. Indicated are ΔC_t_ values normalized to β-actin levels in the HPV-16^+^ mouse cell line C3 (top panel) or the human cell line SiHa (bottom panel). The absence of bars for some splice donors or acceptors denotes undetermined *trans*-splicing. Mean ± SEM (n = 3). (C) alamarBlue cell viability assay with 3′ and 5′ ER constructs using SiHa cells and C3 cells, as well as HPV-18^+^ HeLa cells and HPV^−^ HepG2 cells. Cells were treated with 100 μM GCV for 6 days. Mean ± SEM (n = 3). Significance was tested using two-way ANOVA with Bonferroni post hoc test compared to mock. *p < 0.05, **p < 0.01, ***p < 0.001, and ****p < 0.0001. See also Figure S13.

The design of our tsRNA enables replacement of the BD to target any pre-mRNA of interest. As a second clinically relevant target, we chose HPV-16, which establishes productive infections in basal keratinocytes of the mucous membranes, causing premalignant lesions and cancer. Before cell transformation, HPV-16 DNA integrates into the host cell genome. Beyond surgical removal, the selective destruction of the transduced cells by suicide gene therapy may represent a novel curative approach. In HPV infections, alternative splicing generates multiple isoforms of viral mRNA. We computationally selected the five least structured antisense BDs of 46 to 82 nt in length (mBDE6, mBDE1a, mBDE1b, mBDE2, and mBDE5), which can be directed against HPV-16 transcripts targeting the early viral genes E6, E1, E2, and E5 (Figure 8A).^33^, ^34^ Selected BDs were furnished with target mismatches and cloned into the parental vectors using spacers. Each of the resulting 5′ or 3′ ER tsRNAs can recruit multiple alternative viral A or D sites, which were either published^35^, ^36^ or predicted by us using the ss predictor algorithm Alternative Splice Site Predictor (ASSP).^35^ We monitored recruitment of these ss for *trans*-splicing in the HPV-16-transformed cell lines SiHa (human, 2 viral genome copies) and C3 (murine, multiple truncated and complete viral genome copies)^32^ using real-time RT-PCR (Figure 8B). In both cell lines, HPV-16 transcripts were found to be highly abundant. The cell killing potential of the 5′ ER vectors and the most active 3′ ER constructs E1 and E6 were investigated using the alamarBlue assay. Cell death was selectively triggered in HPV-16-transformed cell lines SiHa and C3, but not in HPV-18-transformed HeLa cells or in the HPV-negative cell line HepG2 (Figure 8C; Figure S13). In SiHa cells, all tsRNAs triggered cell death to the same extent as the positive control; in C3 cells, 3′ ER was more efficient than 5′ ER.

## Discussion

We used computational RNA secondary structure design to generate advanced tsRNAs exhibiting improved activity and specificity in a gene-directed suicide prodrug therapy approach. Emphasis was placed on the design of the target BDs. Earlier studies did not consider RNA structure and concluded that longer BDs are more suitable than shorter BDs. We demonstrated that relatively short BDs of about 50 nt in length can be efficient, minimizing the risk of BD-mediated off-targeting. Different rules were found to govern the design of tsRNA for 3′ and 5′ ER. 3′ ER activities inversely correlated with the degree of BD structure formation; i.e., least structured BDs were most active (Figure 2C). All BDs of this comparison were of similar length, BDs 3mBDopen and 3mBDstruc1 were partly overlapping, and the target binding sites of 3mBDopen and 3mBDopen-inv were even identical. Thus, the observed differences in activity were independent of BD length, target binding site, or distance to the D and can fully be assigned to BD structure. These observations comply with earlier studies on asRNA, which demonstrated that short unstructured asRNA was the fastest binding to its target and triggered the strongest target gene knockdown.^37^, ^38^ Altogether, our findings suggest that unstructured BDs facilitate target binding, which is assumed to be the rate-limiting step in 3′ ER. 5′ ER activities of tsRNAs comprising BD 5mBDopen correlated with the thermodynamic stability of the RNA 3′ end downstream of the BD but did not depend on the presence of a poly(A) tail (Figures 3C–3E). Conversely, for tsRNAs in which different BDs directly constituted the RNA 3′ ends, 5′ ER activities appeared to be virtually independent of BD or 3′ end structure (Figure 2D). It is reasonable to assume that 3′ end stability is important for all 5′ ER constructs. Thus, for 5′ ER tsRNAs, we hypothesize that unstructured BDs trigger faster target binding but are more prone to degradation, whereas structured BDs are slower target binding but more stable, and that the effect of target binding is just balanced against the impact of endogenous RNA stability. We demonstrated that BDs harboring central 2 nt target mismatches significantly enhanced 3′ ER, but not 5 ′ER, activities (Figure 2), though all investigated pairs of RNAs with cBDs and mBDs gave rise to exactly 35 A-to-I-editable positions in the expected BD-target duplexes. These findings indicate that mismatches do not corrupt BD function but instead can exhibit positive effects. Assuming that editing of the cBD-target duplexes impaired 3′ ER activities, this effect held off in the case of 5′ ER and/or was neutralized by other events. The relatively high activity of p-5cBDopen or p-5cBDopen-inv compared with p-5mBDopen or p-5mBDopen-inv might be attributed to a stabilizing structural change or the restoration of a nucleation site, respectively. BD structure design and implementation of target mismatches improved 3′ ER activity about 5-fold each. The combination of both exhibited synergistic (∼30-fold), rather than additive, effects, showing that these two features facilitate *trans*-splicing via different, independent mechanisms.

We investigated the specificity of RNA *trans*-splicing. sp-on-ts, i.e., splicing toward the intended target and ss, is known to compete with *cis*-splicing and off-ts. Here we revealed that specific *trans*-splicing strongly competes with alt-on-ts, which may employ any unintended target ss. Substantial differences were observed between 3′ and 5′ ER specificities. Although alternative and specific *trans*-splicing toward the mini-gene pre-mRNA were found to occur equally frequent in the case of 5′ ER, alt-on-ts was two orders of magnitude more prevalent in 3′ ER. This can be explained by the constellation of available D and A sites: the strong ss of the tsRNAs predominantly *trans*-splice toward strong target ss, which have weak *cis*-splice partnering sites. In addition, the dynamics of transcription and splicing have to be considered. Splicing mainly occurs cotranscriptionally,^39^ implying that on the nascent target RNA 5′ proximal target ss can be recruited earlier for *trans*-splicing than 3′ proximal sites. Splice donor D of the 5′ ER constructs competes with AFP mini-gene donors D3 or D5 for the splice acceptors A3 or A5 in a specific or alternative *trans*-splice reaction (Figure 4A). Analogously, splice acceptor A of the 3′ ER constructs can compete with target acceptors A5 or A3 for the splice donors D5 or D5 during specific or alternative *trans*-splicing, respectively. The efficiency of a potential 5′ or 3′ ER reaction can be estimated by comparing the quotients *D*_ts_*A*_n_/*D*_n_*A*_n_ = *D*_ts_/*D*_n_ or *A*_ts_*D*_n_/*A*_n_*D*_n_ = *A*_ts_/*A*_n_, wherein *D* or *A* represent the Splice Site Prediction (SSP) or ASSP scores predicted for splice donor D or acceptor A sites of the tsRNA (ts) or a target intron (n). The higher these quotients, the more efficient the respective *trans*-splice reaction. For our test system, we calculated *D*_ts_/*D*_5_ > *D*_ts_/*D*_3_ and *A*_ts_/*A*_3_ ≫ *A*_ts_/*A*_5_, indicating that the high frequency of alt-on-ts can partly be explained by the predicted strengths of the involved ss. To fully understand alternative *trans*-splice activities, including the activity of the BD^−^ constructs, the dynamics of competing *cis*- and *trans*-splice reactions have to be considered. Intron 3 is transcribed before intron 5. Thus, the 5′ ER tsRNAs can bind to the nascent target transcript, block *cis*-splicing of intron 3, and recruit either the weak splice acceptor A3 immediately or the stronger acceptor A5 as soon as transcription is complete. BD^−^ 5′ ER RNAs exhibited reduced specific and alternative *trans*-splice activities, indicating the BDs mediated target specificity. Conversely, in the case of 3′ ER, alt-on-ts was highly abundant, and neither alternative nor specific *trans*-splice activities were impaired in the BD^−^ constructs. Hence, the activity of the parental 3′ ER construct was virtually independent of the BD and/or its target binding site, showing that *trans*-splicing to splice donor D3 was already initiated before intron (I) 5 was transcribed, favoring the alternative over the specific *trans*-splice reaction. We speculate that *trans*-splicing triggered by the BD(–) constructs, which were not intentionally featured with a target BD, was facilitated by accidental complementarities between the tsRNAs and the target (Table S2). Number and length of such accidental complementarities correlate with the length of the tsRNA and its target and are difficult to avoid, because all parts of the tsRNA are functionally relevant and cannot simply be removed.^13^ However, a secondary BD shielding the ss of the tsRNA was demonstrated to substantially reduce BD-independent *trans*-splicing (Figure 5). BD-independent *trans*-splicing also implies a risk of off-ts, though we couldn’t detect off-ts toward abundant cellular transcripts.

Most human pre-mRNAs harbor multiple introns. Considering the high frequency of alt-on-ts, only the first D or the last A can specifically be targeted using 3′ or 5′ ER, respectively. Therefore, alt-on-ts represents a double-edged sword: it renders *trans*-splicing-based repair strategies toward targets with many introns less attractive, because all but the first or last exons need to be encoded by the tsRNA (Figure S14A); conversely, *trans*-splicing-based suicide gene therapy is supported by alt-on-ts, which enhances the target-specific death signal. We demonstrated that equipping tsRNA for 3′ ER with short secondary BDs, which bring the *trans*-splicing sites closer to each other, block alternative target ss, and/or shield the ss of the tsRNA, significantly increased sp-on-ts and/or suppressed alt-on-ts, thereby improving the specificity of the *trans*-splice reaction. While the use of secondary BDs will be imperative for *trans*-splicing-based repair strategies, it might not be essential for a suicide gene therapy approach, because the constructs p-3mBDopen (no secondary BD) and p-3mBDopen-DEF (three secondary BDs) triggered comparable levels of death in normal or AFP-overexpressing cells (Figure 5). off-ts toward selected abundant cellular pre-mRNAs was not detected, and the BD(–) constructs p-5BD(–) or p-5BD(–)-ΔHH triggered substantially lower levels of cell death compared with their AFP-targeting counterparts p-5mBDopen or p-5mBDopen-ΔHH. Compared with the mock, the BD(–) constructs did not induce significant levels of death in cells expressing endogenous AFP levels until days 3 or 4 (Figures 6A and 6B). In addition, our HPV-16-targeting tsRNAs didn’t trigger significant death in HPV-16-negative cell lines (Figure 8C). Finally, work in which we reprogrammed the parental vectors of this study for HIV-1-targeting did not indicate any toxicity triggered by off-target effects.^40^ The cell death signal must reach a cellular threshold level to kill the target cell. In the consequence, low to moderate levels of off-ts, which alone are insufficient to trigger cell death, can contribute to a target cell-specific death signal as long as on-ts elevates the level of cell death signal over the threshold.

Our findings and considerations indicate off-ts poses a relatively low risk. However, more systematic studies, including high-throughput cDNA sequencing analyses, need to be performed before this technology can advance toward clinical testing.

We report use of dual-targeting tsRNAs, which all exhibited a higher suicide potential at the lower drug concentration compared with the conventional single-targeting sequence. That was expected, because *trans*-splicing activity correlates with the overall concentration of target RNA. Hence, suicide tsRNAs targeting multiple disease biomarkers are expected to further improve both activity and specificity for a distinct disease.

The levels of cell death triggered with our constructs were surprisingly high, considering that only a fraction of cells can efficiently be transfected. That might be explained by manifold alt-on-ts events involving alternative target ss (Figure S14B) and/or by a bystander effect that was reported for the HSVTK/GCV system *in vitro* and *in vivo*.^41^, ^42^ Accordingly, the active drug GCV triphosphate can diffuse into healthy non-HSVtk-expressing neighboring cells after release from dying cells. *In vivo*, this effect can be enhanced by a distant bystander effect, in which dying cells can induce host immune responses mediated by natural killer cells and T cells.^43^

In summary, we demonstrated BD design represents the key to improve both activity and specificity of tsRNA. As a guideline, we suggest tsRNA for suicide gene therapy should capitalize on copious alt-on-ts toward one or more suitable pre-mRNA targets. Accordingly, it should comprise unstructured BDs of 50 to 100 nt in length imperfectly binding to 3′ or 5′ proximal introns of one or multiple biomarker pre-mRNAs for 3′ or 5′ ER, respectively. A secondary BD internally shielding the *trans*-ss may be considered to suppress off-ts. BDs of tsRNA for 5′ ER should be followed downstream by a stable RNA secondary structure (Figure S15). The success of gene therapy approaches is hampered by the delivery problem, and delivery issues might limit *in vivo* applications of our suicide vectors. However, high *trans*-splice activities can partly compensate for low delivery efficiency. Furthermore, it can be regarded as an advantage that cell death can already be triggered via one-time delivery of the suicide sequences using non-integrating transient gene expression vectors, including DNA mini-circles or dumbbell-shaped DNA minimal vectors.^44^, ^45^ The structurally optimized second-generation suicide RNAs reported here may be explored for curative topical non-invasive treatment, e.g., by applying a cream or gel onto premalignant or cancerous HPV-16-associated lesions of the skin or mucous membranes. For HCC targeting, suicide vectors covalently linked with triantennary N-acetylgalactosamine residues (GalNAc_3_) can be applied to patients subcutaneously, to the portal vein of the liver, or intra-tumorally.^46^ Alternatively, the suicide RNAs may be reprogrammed by replacing the BDs to target other diseased cell types that are characterized by high expression levels of one or multiple disease-specific biomarkers either *ex vivo* or *in vivo*.

## Materials and Methods

### Experimental Design

The aim of this study was to explore RNA structure design toward *trans*-splicing-based suicide RNA with enhanced on-target activity and specificity. As targets, we selected human AFP-expressing HCC-derived cells, as well as HPV-16-transformed cells. The activity and specificity of *trans*-splicing was genotypically monitored using real-time RT-PCR and sequencing. *trans*-splicing triggered death of target cells was monitored using the alamarBlue cell viability assay, the Annexin V/PI apoptosis assay, and the comet assay. For the comet assay, tail moments above the third quarticle (Q3) + 1.5 (interquartile range [IQR]) are shown as dots (outliers). Cell lines HEK293T and HepG2 were purchased from ATCC; cell lines CL48, PLC/PRF5, SNU495, Hep3B, Sk.Hep1, C3, SiHa, CaSki, and HeLa were donations as acknowledged. Sample size and replicates are specified in each figure legend.

### Computational Selection of Target BDs

The software foldanalyze^24^ was used to select short unstructured cBDs of 50 in length within the complete asRNA structure space that can be directed against all introns of the AFP or alternative pre-mRNAs. Exons were excluded from the BD selection process to avoid interference with the cytoplasmic RNA-silencing machinery, because exon-targeting BDs could trigger formation of double-stranded RNA in the cytoplasm. Unstructured cBDs were selected to harbor a maximum number of external bases (terminal-free nucleotides) and to have a high Gibbs free folding energy. Structured cBDs were selected using foldanalyze to harbor a minimum number of external bases, to have a low Gibbs free folding energy, and to bind the nearby target or to overlap with the unstructured cBD binding sites. A subset of cBDs was furnished with target mismatches to generate mBDs, which were selected not to alter the BD structure. Structures of the cBDs and mBDs were confirmed by RNA secondary structure (minimum free energy and centroid) predictions using the software tools mfold and RNAfold*.*^26^, ^27^

### *trans*-Splicing Vector Design

The 3′ ER *trans*-splicing constructs consisted of a cytomegalovirus (CMV) promoter (pEGFP-N1, Clontech, NCBI Nucleotide: U55762) followed by a target BD. BDs were linked to the tsRNA via spacers, which were designed to preserve the selected BD structures. The splicing domain was composed of an intronic splice enhancer (ISE),^47^, ^48^ a branchpoint (BP)^18^ 5′-TACTAACA-3′, a polypyrimidine tract (PPT)^49^, ^50^ 5′-TTTTTTTCCCTTTTTTTCC-3′, and a splice acceptor AG/G. The coding domains was composed of the P2A^51^ proteolytic cleavage site followed by the HSVtk coding sequence (cds) (NCBI Nucleotide: AF057310). The HSVtk gene was devoid of a start codon and could only be translated after *trans*-splicing employing the translational start of the target message. The HSVtk gene was furnished with an A/G-rich ESE^52^ generated by using degenerative alternative codons without altering the HSVtk amino acid sequence (Figure S1A). A β-globin mini-intron of 133 bp (pCMVTNT, NCBI Nucleotide: AF477200.1) was introduced in a CAG/G motif of the HSVtk gene to generate a splice donor (CAG/GT) and acceptor (AG/G) consensus motif. The coding domain was followed a SV40 poly(A) site (pcDNA3.1, Life Technologies). *trans*-splicing constructs for 5′ ER were composed of the same molecular features as described earlier for 3′ ER constructs but in a different order and with variations. The CMV promoter was followed by a translational signal motif, including the original cap site of the AFP gene^53^ and the consensus Kozak sequence 5′-GCCRGCCAUGG-3′.^54^, ^55^ The translational start codon was continued with the HSVtk coding domain, including ESE and mini-intron, followed by a splice donor^56^ 5′-CAG/GTAAGTAT-3′ and the ISE. The adjacent BD was linked via a spacer to preserve the selected BD structure and followed downstream by a tertiary structure-stabilized, *cis*-cleaving HHRz.^23^ Correct folding of the active ribozyme structure was supported by inserting a spacer between the ribozyme and the poly(A) site. As controls, we designed constructs for 3′ or 5′ ER with a ΔHSV harboring two point mutations: an A-to-G mutation at position 115 (glycine to glutamic acid) and a G-to-A mutation at position 649 (histidine to arginine).^57^ The ΔHSV produces a partly inactive full-length protein. ss mutants for 3′ ER were prepared by mutating the A site from AG to TC; in addition, the BP was mutated from A to C (Δss1) or 6 of 8 positions of the ss, including the BP (Δss2). ss mutants for 5′ ER were prepared by mutating either 7 of 11 positions of the ss but maintaining the D GT motif (Δss1) or by changing 10 of 11 positions, including a GT to AC mutation of the SD (Δss2). To block HHRz cleavage, the consensus cleavage motif was changed from GUC to ACC in the ΔHH 5′ ER constructs.

### Plasmid Construction

In total, 80 constructs were generated. The parental 3′ and 5′ ER constructs p-3mBDopen and p-5mBDopen were generated by gene synthesis (GeneArt, Regensburg) and cloned into pVAX1 (Addgene) using *Spe*I and *Bbs*I. The parental vectors served as master vectors for sub-cloning of all other *trans*-splicing constructs (Supplemental Materials and Methods).

### Cell Culture

Cell lines were purchased from ATCC (HEK293T and HepG2) or were donations (CL48, PLC/PRF5, SNU495, Hep3B, Sk.Hep1, C3, SiHa, CaSki, and HeLa). Cells were maintained at 37°C in a humidified incubator with 5% CO_2_ in DMEM or RPMI 1640 (HyClone, Thermo Scientific), supplemented with 10% fetal bovine serum (HyClone) and 1% penicillin-streptomycin. The cells were passaged every 3–4 days.

### Transfection of Cells

HepG2 cells were transfected with ∼70%–90% confluency in 6-well plates for western blotting, 12-well plates for fluorescence-activated cell sorting (FACS) analyses, and in 24-well plates for all other analyses using either Lipofectamine 3000 (for FACS studies only) or Lipofectamine 2000 (Life Technologies) according to the manufacturer’s protocol. In 24-well plates, cells were (co)transfected with 1 μg μ total DNA: either 500 ng of *trans*-splicing vector and 500 ng of AFP mini-gene for AFP overexpression studies or 1 μg of *trans*-splicing constructs to study *trans*-splicing toward the endogenous target. In 12-well or 6-well plates, cells were transfected with 2 or 4 μg of DNA, respectively. For flow cytometry analyses of egfp-negative *trans*-splicing vectors, 500 ng of pEGFP-C2 plasmid was cotransfected, along with the other constructs.

### RNA Isolation

Total RNA was isolated 24 hr post-transfection using the RNeasy plus kit (QIAGEN) following the manufacturer’s protocol. RNA concentrations were measured using NanoDrop 2000.

### cDNA Synthesis and Real-Time RT-PCR

500 ng of RNA was converted to cDNA using the First Strand SuperScript RTIII (Invitrogen) kit with 200 ng of random hexamers and 10 μM of dinucleotide triphosphates (dNTPs). The reaction conditions were 25°C for 5 min, followed by 50°C for 2 hr and enzyme inactivation at 70°C for 15 min. 20 ng of cDNA was used as the template for real-time RT-PCR. TaqMan quantification of the cDNAs was performed in ABI 7900HT using specific probe and primer sets for the detection of *cis*- and *trans*-splicing. One set of probes were specific for AFP target regions, i.e., AFP-probe-exon-5 and AFP-probe-exon-4 to detect *cis*- and *trans*-splicing in the 3′ and 5′ ER experiments, respectively. Together with the AFP probe, primers FP-afp-exon-5(set1) and RP-afp-exon-6 were used to detect *cis*-splicing and primers FP-afp-exon-5(set1) and RP-HSVtk were used to detect *trans*-splicing in the 3′ ER experiments. Analogously, primers FP-afp-exon-3 and RP-afp-exon-4(set1) were used to detect *cis*-splicing and primers FP-HSVtk(set1) and RP-afp-exon-4(set1) were used to detect *trans*-splicing in the 3′ ER experiments. For 3′ ER detection, an additional amplicon comprising HSVtk-probe(3′ER) and primers FP-afp-exon-5(set2) and RP-HSVtk was used. For 5′ ER detection, the additional amplicon comprising HSVtk-probe(5′ER) and primers FP-HSVtk(set2) and RP-afp-exon-4(set2) was used. To detect *trans*-splicing toward the overexpressed or endogenous target message, 40 or 50 PCR cycles were used, respectively. Sequences of primers and probes are listed in Table S3.

### PCR

For detection of *cis*- and *trans*-splicing, cDNA samples were amplified using two-step PCR (30 + 30 or 35 + 35 cycles) and *Taq* DNA polymerase (Fermentas), and products were analyzed by 1% agarose gel electrophoresis. In 3′ ER experiments, *cis*-spliced and unspliced RNA was detected using primers FP-afp-exon-5(set1) and RP-afp-exon-6; *trans*-spliced RNA was detected using primers FP-afp-exon-5(set1) and RP-HSVtk. In 5′ ER experiments, *cis*-spliced and unspliced RNA was detected using primers FP-afp-exon-3 and RP-afp-exon-4(set1); *trans*-spliced RNA was detected using primers FP-HSVtk(set1) and RP-afp-exon-4(set1). To detect specific versus alt-on-ts, cDNAs from 3′ ER experiments involving constructs p-3mBDopen and p-3BD(–) were amplified using primers FP-afp-exon-5(set2) and RP-HSVtk to detect sp-on-ts or primers FP-afp-exon-3 and RP-HSVtk for alt-on-ts detection. Analogously, cDNA from 5′ ER experiments involving constructs p-5mBDopen and p-5BD(–) were amplified with primers FP-HSVtk(set2) and RP-afp-exon-4(set2) to detect sp-on-ts or primers FP-HSVtk(set2) and RP-afp-exon-6 for alt-on-ts detection.

### Alamar Blue Assay

Prodrug GCV (Sigma) was added to the cells at concentrations of 10 μM or 100 μM 24 hr post-transfection. The alamarBlue cell viability reagent (Thermo Scientific) was added 24 hr after GCV treatment, and fluorescence was measured at 230 nm/290 nm (excitation/emission) after an incubation time of 90 min at 37°C. Subsequently, media and drug were replenished and measurements were repeated for 6 consecutive days. Positive and negative assay controls were designed according the manufacturer’s protocol.

### Comet Assay

100 μM of GCV was added to HepG2 cells 24 hr post-transfection. 24 hr after drug treatment, cells were harvested using alkaline lysis.^58^ Single-cell electrophoresis was performed, and comets were stained with 10 μg/mL PI (Life Technologies) and analyzed under a fluorescent microscope at 10× and 20× magnifications. No significant heterogeneity in DNA damage was observed. 150 comets were scored per sample, which is three times more than recommended by Olive and Banáth.^58^

### Annexin V/PI Apoptosis Assay

HepG2 cells were treated with 100 μM of GCV 24 hr post-transfection. Cells were harvested 48 hr after GCV treatment and stained with PI and Alexa Fluor 647 Annexin V (Life Technologies) in annexin binding buffer according to the manufacturer’s protocol. Apoptosis was monitored in EGFP^+^ live cells using flow cytometry, and the percentage of apoptotic cells was indicated as (early and late apoptosis + GCV) − (early and late apoptosis − GCV).

### Western Blotting

Cells were harvested 24 hr post-transfection, and 50 μg of total protein was analyzed on 10% SDS-PAGE, transferred on a polyvinylidene fluoride (PVDF) membrane, and blocked with 5% milk (w/v). Respective primary antibodies (HSVTK vL-20 goat polyclonal, Santa Cruz, Cat. No. sc-28038; AFP goat polyclonal, Santa Cruz, Cat. No. sc-8108; and β-actin rabbit polyclonal, Santa Cruz, Cat. No. sc-130656) were incubated in 5% milk overnight at 4°C, followed by 2 hr incubation with secondary antibodies (HSVTK and AFP anti-goat, Santa Cruz, Cat. No. sc-2020, and β-actin anti-rabbit, Santa Cruz, Cat. No. sc-2357). Chemiluminescence readings of the blots were done using Pierce enhanced chemiluminescence (ECL) western blotting substrate (Thermo Scientific) and an imager (Bio-Rad).

### ss Prediction

The strength and nature of splice sites were predicted using the software ASSP^35^ (http://wangcomputing.com/assp/overview.html) and Berkeley Drosophila Genome Project (BDGP) SSP^59^ (http://www.fruitfly.org/seq_tools/splice.html), applying default cutoff values. To predict the nature of splice sites in HPV-16 sequences, ASSP was used to identify constitutive or cryptic splice acceptors and donors based on the overall score and confidence. Besides reported splice sites,^56^ alternative splice sites with confidence > 0.89 and score > 5.5 or constitutive splice sites with confidence > 0.1 and score > 7.7 were investigated with regard to *trans*-splicing.

### Statistical Analysis

Error bars are ±SEM of three independent experiments. One-way ANOVA was used for the comparison of mean values of three or more samples with Tukey post hoc test. Two-way ANOVA was used for comparison of mean values of three or more samples with two categorically independent variables. Bonferroni or Tukey post hoc tests were used. Prism 6 GraphPad software was used for the statistical analyses. *p < 0.05, **p < 0.01, ***p < 0.001, and ****p < 0.0001.

## Author Contributions

V.P., J.E., and S.P. developed the concept. V.P. and S.P. designed the experiments. S.P., P.S.L., Z.H.O., and F.O. carried out the experiments, and all authors analyzed the data. V.P. and S.P. wrote the manuscript.

## Conflicts of Interest

The authors declare competing financial interests: pending patent application UK 1605586.5 (2016) / PCT/SG2017/050183 (2017).

## Supplemental Information

Document S1. Supplemental Materials and Methods, Figures S1–S15, and Tables S1 and S2

Table S3. List of Oligonucleotides, Probes, and Primers

Document S2. Article plus Supplemental Information
