# Supplementary material for: RNA Structure Design Improves Activity and Specificity of trans-Splicing-Triggered Cell Death in a Suicide Gene Therapy Approach
Source: Mol Ther Nucleic Acids. 2018 Jan 31;11:41–56. doi: 10.1016/j.omtn.2018.01.006 (PMC5849863; doi:10.1016/j.omtn.2018.01.006)
Supplement: Document S1. Supplemental Materials and Methods, Figures S1–S15, and Tables S1 and S2 [file mmc1.pdf]

OMTN, Volume *11*

Supplemental Information

RNA Structure Design Improves Activity and Specificity of *trans*-Splicing-Triggered Cell Death in a Suicide Gene Therapy Approach

Sushmita Poddar, Pei She Loh, Zi Hao Ooi, Farhana Osman, Joachim Eul, and Volker Patzel

Supplemental Information

Table of Contents:

A, Supplemental Figures

Figure S1. *Trans*-splicing RNAs harbouring an improved HSVtk gene trigger the formation of chimeric RNA and HSVtk expression

Figure S2. RNA secondary structures of 3'ER constructs and BDs

Figure S3. RNA secondary structures of 5'ER constructs and BDs

Figure S4. Introduction of a customized spacer sequence

Figure S5. Relative *trans*-splicing activities of 3' & 5'ER tsRNAs harbouring structured BDs

Figure S6. RNA secondary structure predictions of 5'ER tsRNA with 3' end modifications

Figure S7. Relative *trans*-splicing activities of BD-negative constructs and corresponding splice site mutants

Figure S8. Death of AFP expressing HepG2 cells triggered by *trans*-splicing toward the over- expressed or endogenous AFP message

Figure S9. Annexin V/PI apoptosis assay of HepG2 cells co-transfected with *trans*-splicing vectors, AFP mini-gene, and pEGFP

Figure S10. Gating strategy for the annexin V/PI apoptosis assays of HepG2 cells transfected with EGFP-expressing *trans*-splicing vectors

Figure S11. Comet assay in HepG2 cells co-transfected with *trans*-splicing vectors and AFP mini-gene

Figure S12. Cell death triggered by *trans*-splicing of dual-targeting tsRNAs toward two endogenous pre-mRNA targets

Figure S13. Cell death triggered by HPV-16-targeting trans-splicing vectors at 10 µM, 100 µM or no GCV treatment

Figure S14. Alternative splice site targeting

Figure S15. Schematic drawing highlighting recommendations for the rational design of *trans*-splicing based suicide vectors

B, Supplemental Tables

Table S1. List of Constructs

Table S2. Accidental complementarity between BD-negative tsRNA and the AFP mini-gene message

Table S3. List of oligonucleotides, probes, and primers

C, Supplemental Materials and Methods

D, Supplemental References

A, Supplemental Figures

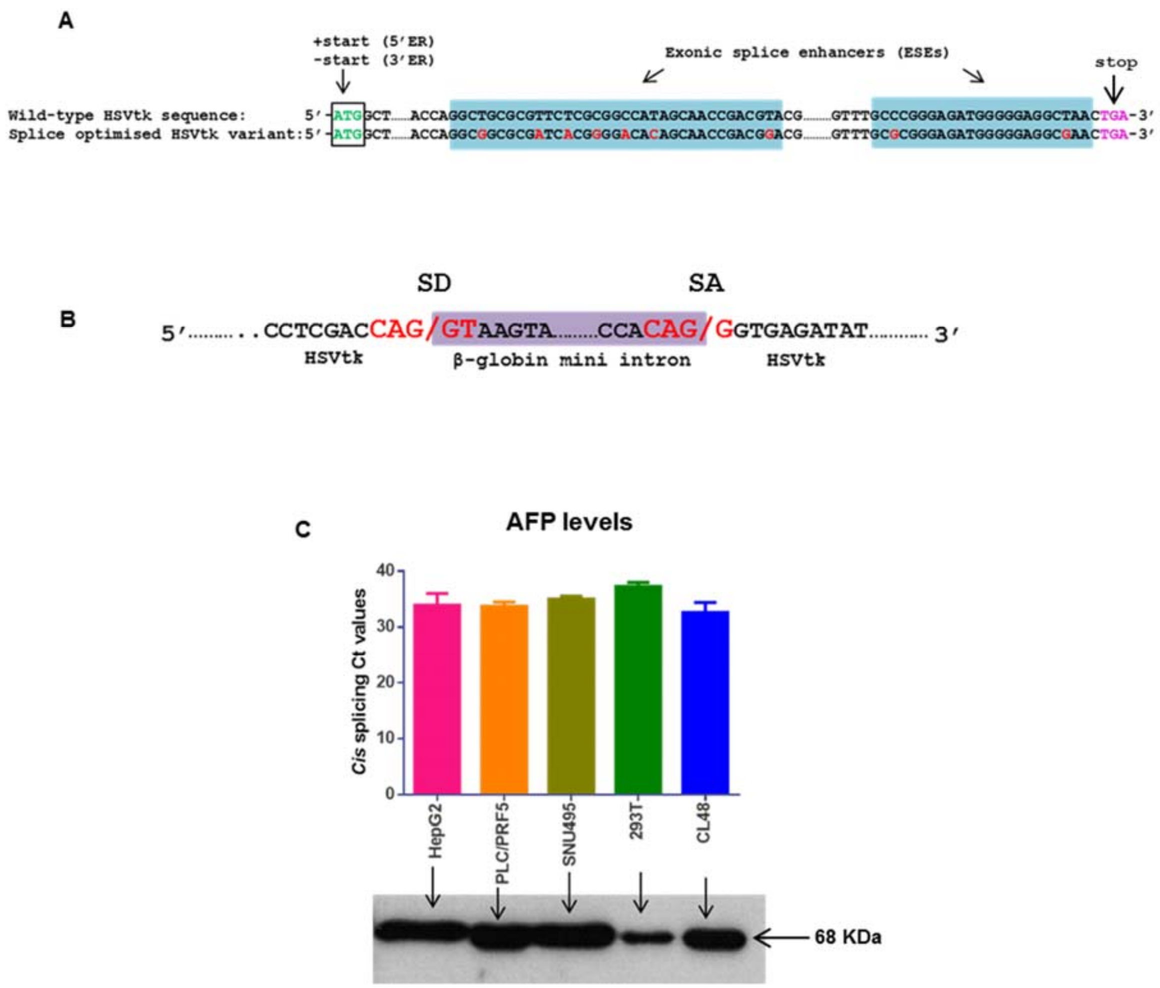

D

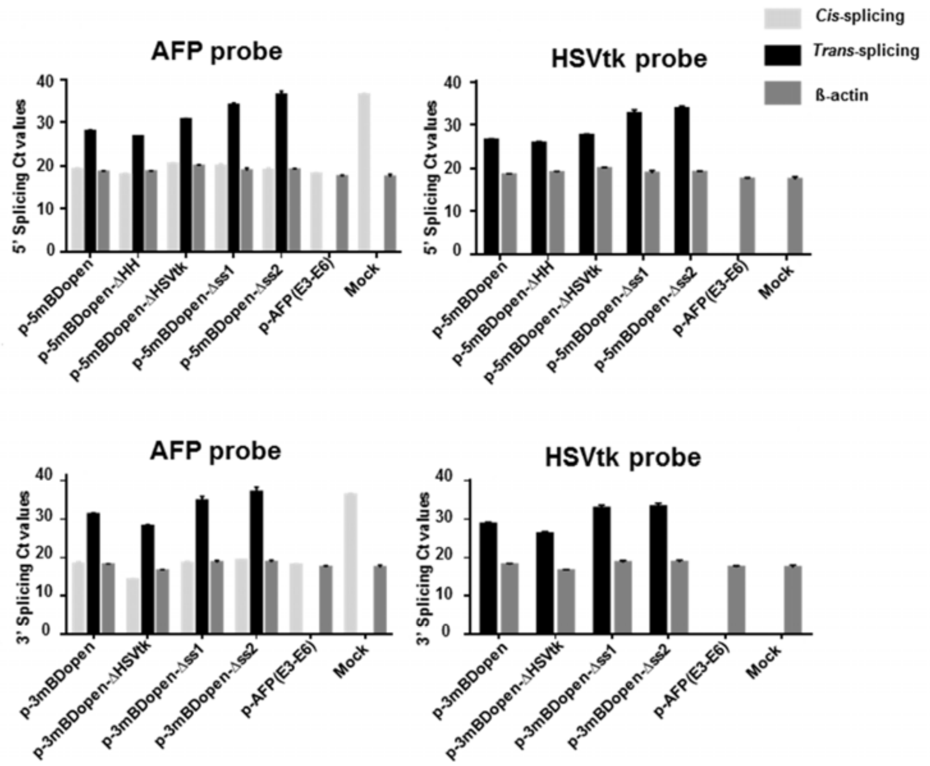
Cis-splicing
Trans-splicing
ß-actin
AFP probe
HSVtk probe
5' Splicing Ct values
3' Splicing Ct values
0
10
20
30
40
p-5mBDopen
p-5mBDopen-ΔHH
p-5mBDopen-ΔHSVtk
p-5mBDopen-Δss1
p-5mBDopen-Δss2
p-AFP(E3-E6)
Mock
p-3mBDopen
p-3mBDopen-ΔHSVtk
p-3mBDopen-Δss1
p-3mBDopen-Δss2

E

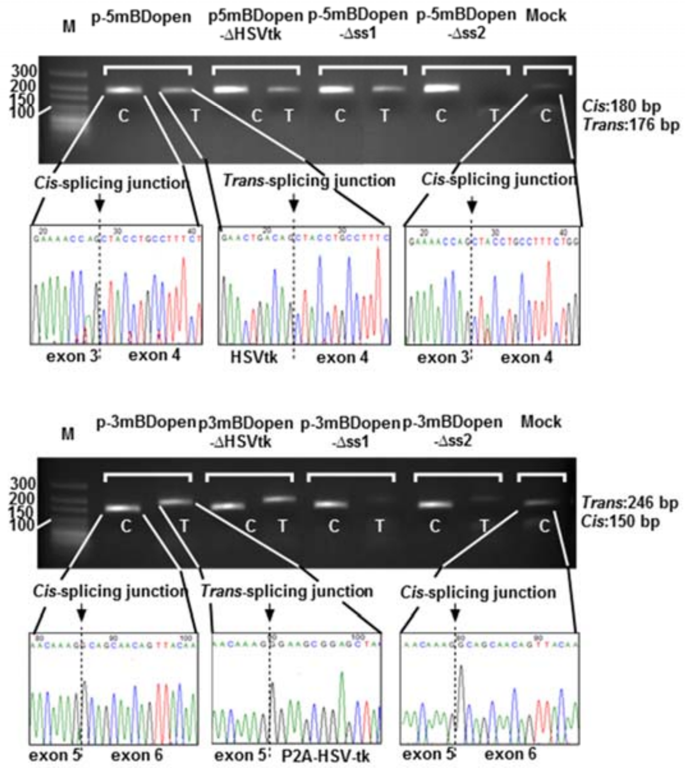
M
p-5mBDopen
p5mBDopen -ΔHSVtk
p-5mBDopen -Δss1
p-5mBDopen -Δss2
Mock
300
200
150
100
C
T
Cis:180 bp
Trans:176 bp
Cis-splicing junction
Trans-splicing junction
exon 3
exon 4
HSVtk
p-3mBDopen
p3mBDopen -ΔHSVtk
p-3mBDopen -Δss1
p-3mBDopen -Δss2
Trans:246 bp
Cis:150 bp
exon 5
exon 6
P2A-HSV-tk

F

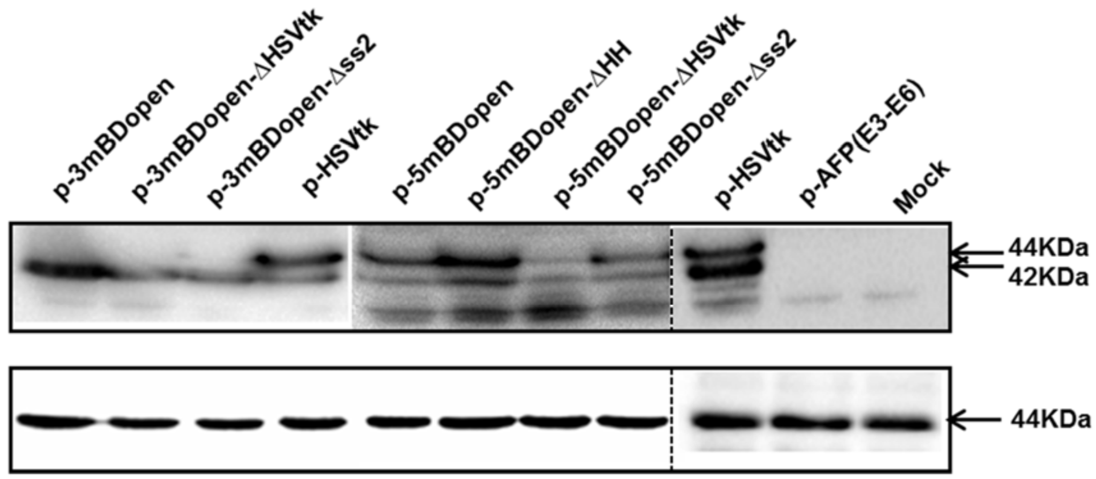
p-3mBDopen
p-3mBDopen-ΔHSVtk
p-3mBDopen-Δss2
p-HSVtk
p-5mBDopen
p-5mBDopen-ΔHH
p-5mBDopen-ΔHSVtk
p-5mBDopen-Δss2
p-HSVtk
p-AFP(E3-E6)
Mock
HSVtk
44KDa
42KDa
ß-actin
44KDa

G

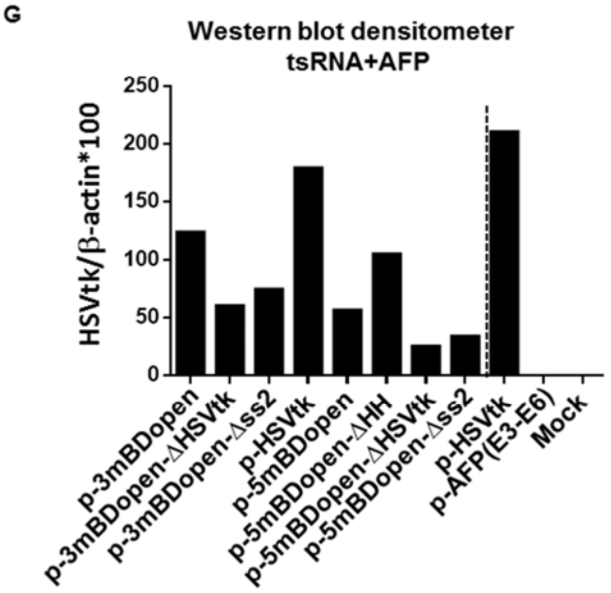
Western blot densitometer
tsRNA+AFP
HSVtk/β-actin*100
250
200
150
100
50
0
p-3mBDopen
p-3mBDopen-ΔHSVtk
p-3mBDopen-Δss2
p-HSVtk
p-5mBDopen
p-5mBDopen-ΔHH
p-5mBDopen-ΔHSVtk
p-5mBDopen-Δss2
p-HSVtk
p-AFP(E3-E6)
Mock

I

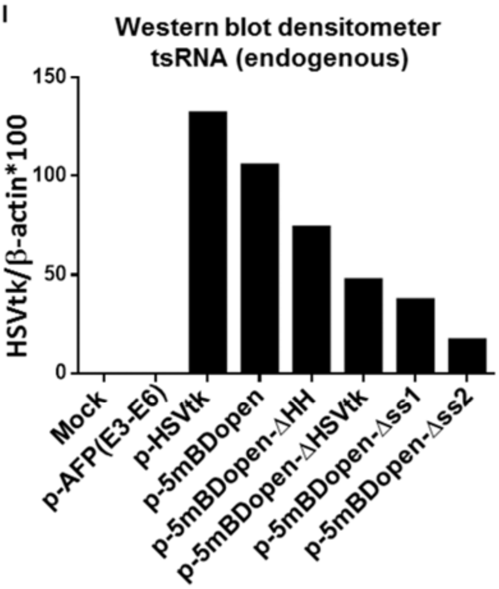
Western blot densitometer
tsRNA (endogenous)
HSVtk/β-actin*100
150
100
50
0
Mock
p-AFP(E3-E6)
p-HSVtk
p-5mBDopen
p-5mBDopen-ΔHH
p-5mBDopen-ΔHSVtk
p-5mBDopen-Δss1
p-5mBDopen-Δss2

H

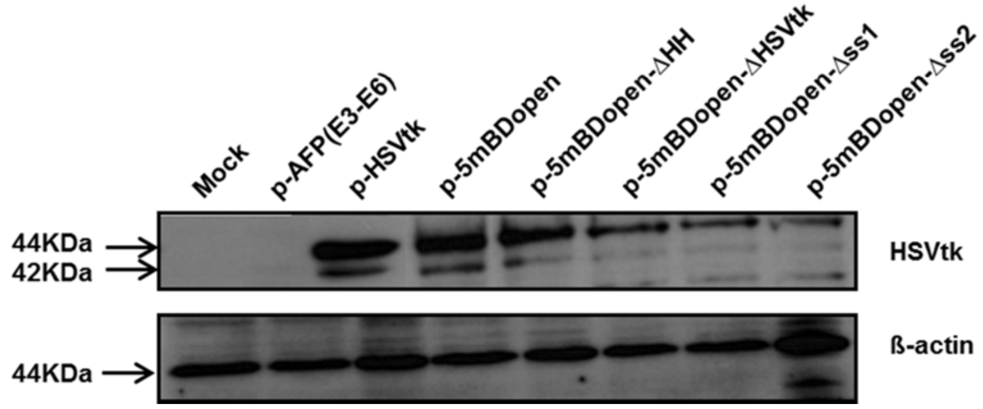
Mock
p-AFP(E3-E6)
p-HSVtk
p-5mBDopen
p-5mBDopen-ΔHH
p-5mBDopen-ΔHSVtk
p-5mBDopen-Δss1
p-5mBDopen-Δss2
44KDa
42KDa
HSVtk
ß-actin
44KDa

Figure S1. *Trans*-splicing RNAs harbouring an improved HSVtk gene trigger the formation of chimeric RNA and HSVtk expression (Referring to Figure 1). (**A**) Introduction of AG-rich exonic splice enhancers (ESEs) into start and end of HSVtk gene by exploring alternative codon usage. Base substitutions are indicated in red. (**B**) Scheme of implementation of the 133 nt beta globin mini-intron in the HSVtk gene generating functional splice donor (SD: CAG/GT) and acceptor (SA: CAG/G) sites. (**C**) rtRT-PCR (upper panel) and western blot (lower panel) detection of AFP expression levels in HCC cell lines and in HEK293T cells. (**D**) rtRT-PCR quantification of *trans*- and *cis*-spliced RNA in HepG2 cells transfected with the AFP-mini gene and *trans*-splicing vectors for 5'ER (top panel) or 3'ER (lower panel). Indicated are raw threshold cycles (C_t) determined using either an AFP-specific (binding exon 4 for 5'ER and exon 6 for 3'ER, left panel) or an HSVtk-specific (binding at 3' terminus for 5'ER and at the 5' terminus for 3'ER, right panel) TaqMan probe. actin beta was used as internal control. Mean ± SEM (n=3). (**E**) Two-step (30+30 cycles) RT-PCR amplification of splice products involving the over-expressed AFP message in the presence of 5'ER (top) or 3'ER (bottom) *trans*-splicing RNA. PCR products were analyzed by 1% agarose gel electrophoresis and splice junctions were confirmed by sequencing. C, *cis*-splicing; T, *trans*-splicing. Mock shows endogenous levels of AFP *cis*-splicing detection (D, left panel) and (E). (**F**) Western blot analyses of HSVtk (upper panel) expression after *trans*-splicing toward the over-expressed AFP mini-gene message. While 3'ER triggered the formation of a 42 kDa HSVtk isoform, 5'ER and the HSVtk positive control (+) lead to the expression of 42 and 44 kDa isoforms. No chimeric AFP-HSVtk fusion proteins were detectable with the 3'ER constructs pointing towards efficient P2A-proteolytic cleavage. In case of 5'ER, the splice site mutants triggered clearly reduced levels of HSVtk expression as compared with the parental construct indicating the vast majority of HSVtk protein originated from *trans*-splicing and not from leaky splicing-independent expression. The negative control (-) shows no HSVtk protein. actin beta (lower panel) was used as a control. (**G**) Densitometry analyses of the HSVtk protein levels from AFP-mini gene expressing cells normalised to actin beta expression. In samples expressing two HSVtk isoforms, both bands were quantified together. (**H**) Western blot analyses of HSVtk (upper panel) after *trans*-splicing toward the endogenous AFP message. Negative controls do not show HSVtk protein whereas the positive control and *tran*s-splicing samples display the two HSVtk isoforms.Actin beta (lower panel) was used as a control. (**I**) Densitometry analyses of the HSVtk protein levels triggered by *trans*-splicing toward the endogenous AFP message normalised with actin beta.

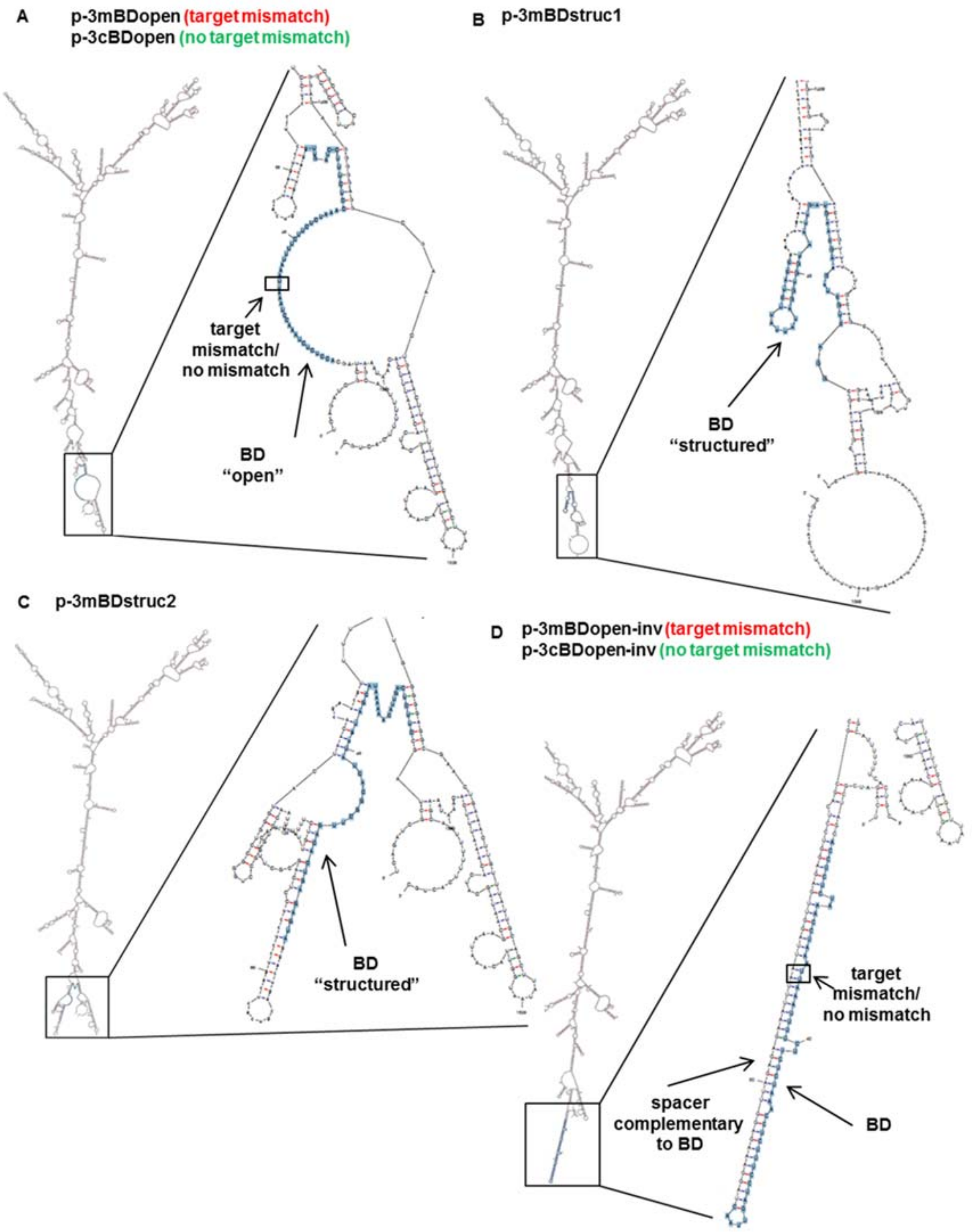

Figure S2. RNA secondary structures of 3'ER constructs and BDs (Referring to Figure 2). (**A**) Structure of parental 3'ER tsRNA p-3m/cBDopen harboring an unstructured BD. This construct was designed with a mismatched mBD or a fully complementary cBD both of which were predicted to fold the same RNA secondary structure. (**B**, **C**) Structures of tsRNAs p-3mBDstruc1 and p-3mBDstruc2 harboring structured mismatched BDs. (**D**) Structure of tsRNA p-3m/cBDopen-inv harboring 3m/cBDopen but fully embedded in a stem-loop structure by an inverted repeat. This construct was designed with a mismatched mBD or a fully complementary cBD both of which were predicted to fold the same RNA secondary structure. RNA secondary structures as predicted by *mfold*. BD sequences are shaded in blue. Boxes indicate sequence positions that differ in the mBD and cBD variants.

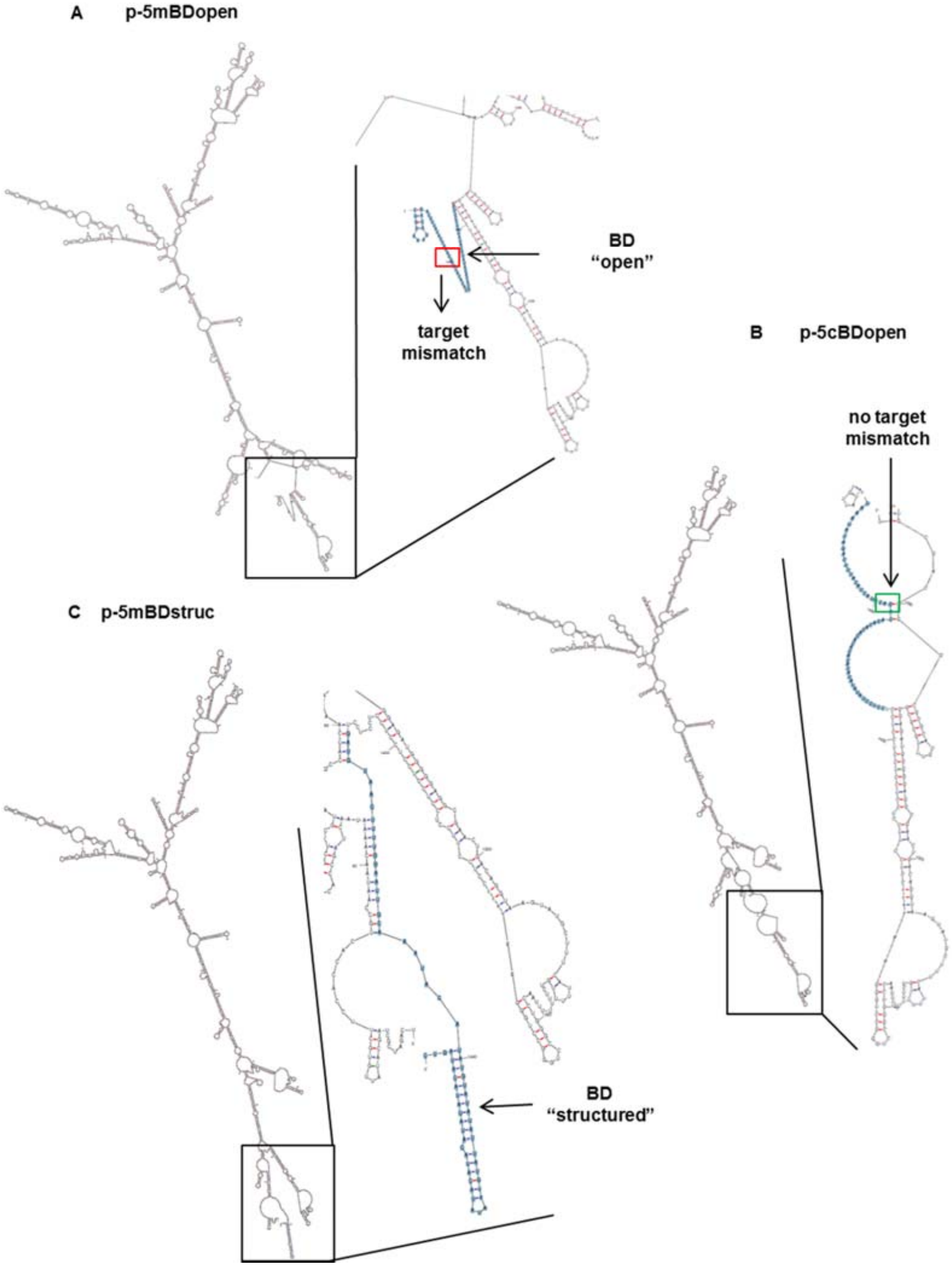
A p-5mBDopen
BD
“open”
target
mismatch
B p-5cBDopen
no target
mismatch
C p-5mBDstruc
BD
“structured”

D p-5mBDopen-inv

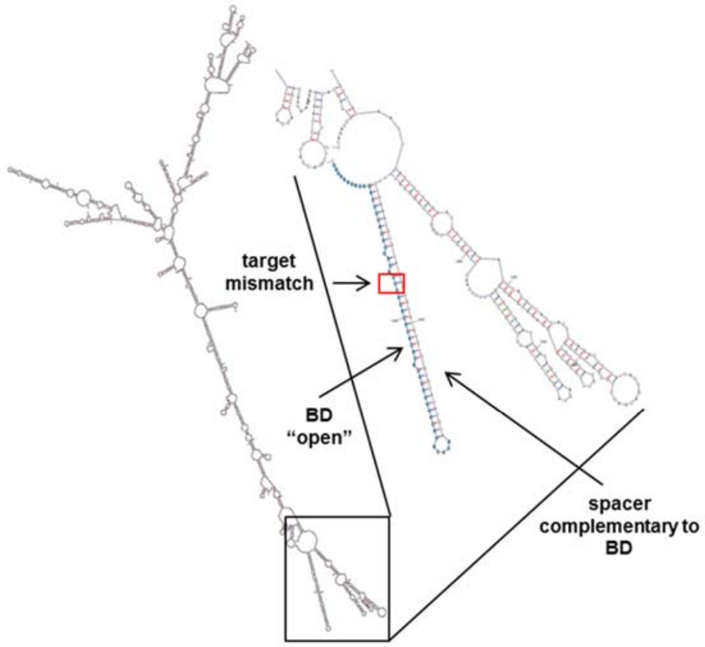

E p-5cBDopen-inv

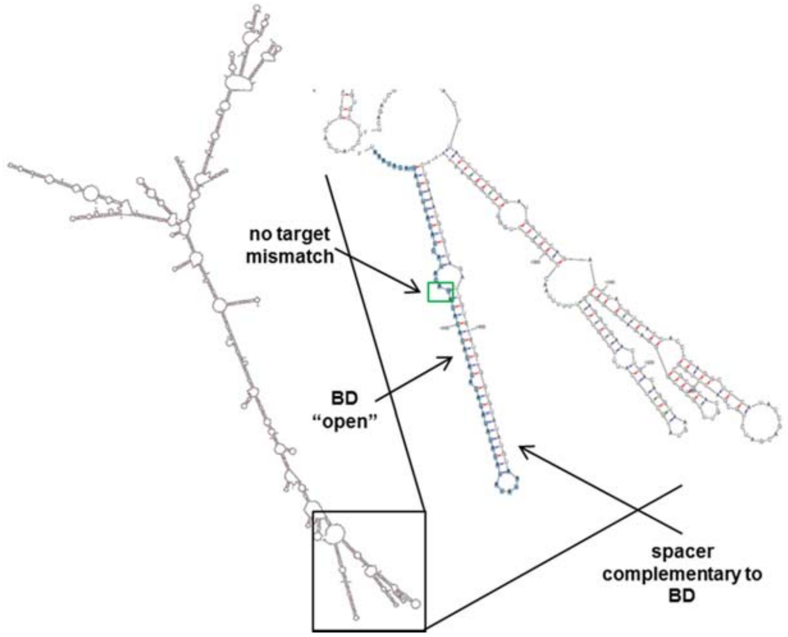

Figure S3. RNA secondary structures of 5'ER constructs and BDs (Referring to Figure 2). (**A**) Structure of parental 5'ER tsRNA p-5mBDopen harboring an unstructured BD with a target mismatch (red box). (**B**) Structure of parental 5'ER tsRNA p-5cBDopen harboring an unstructured BD fully complementary to the target. The corresponding position that was mismatched in p-5mBDopen is indicated (green box). (**C**) Structure of tsRNA p-5mBDstruc harboring a structured mismatched BD. (**D**) Structure of tsRNA p-5mBDopen-inv harboring 5mBDopen but fully embedded in a stem-loop structure by an inverted repeat. (**E**) Structure of tsRNA p-5cBDopen-inv harboring 5cBDopen but fully embedded in a stem-loop structure by an inverted repeat. RNA secondary structures as predicted by *mfold*. BD sequences are shaded in blue. Boxes indicate sequence positions that differ in the mBD and cBD variants.

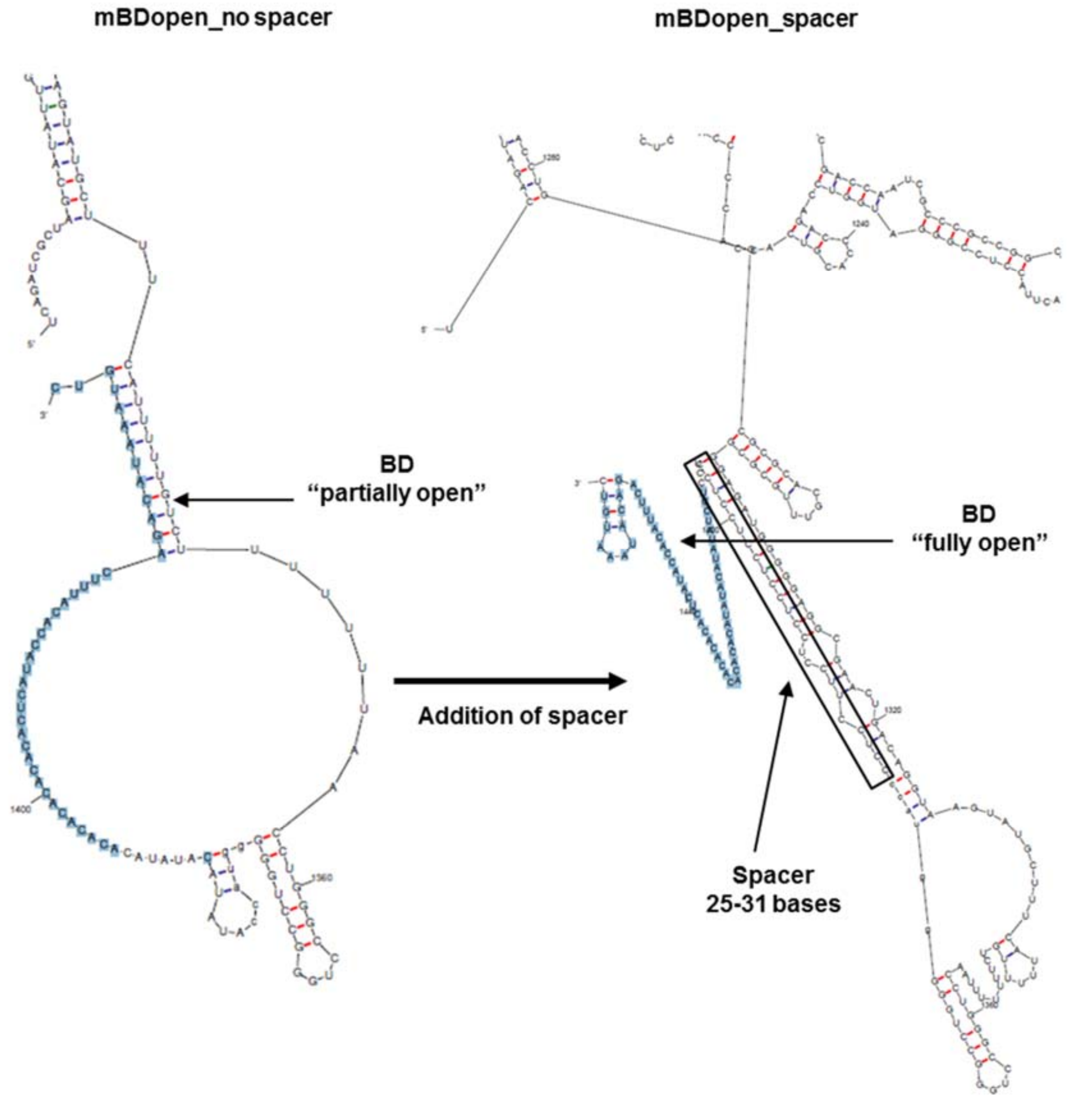

Figure S4. Introduction of a customized spacer sequence (Referring to Figure 2). Spacer were inserted either upstream or downstream of the BD to preserve the *in silico* selected structure (here open structure) upon fusion to the *trans*-splicing RNA (example). The BD sequence positions are shaded in blue.

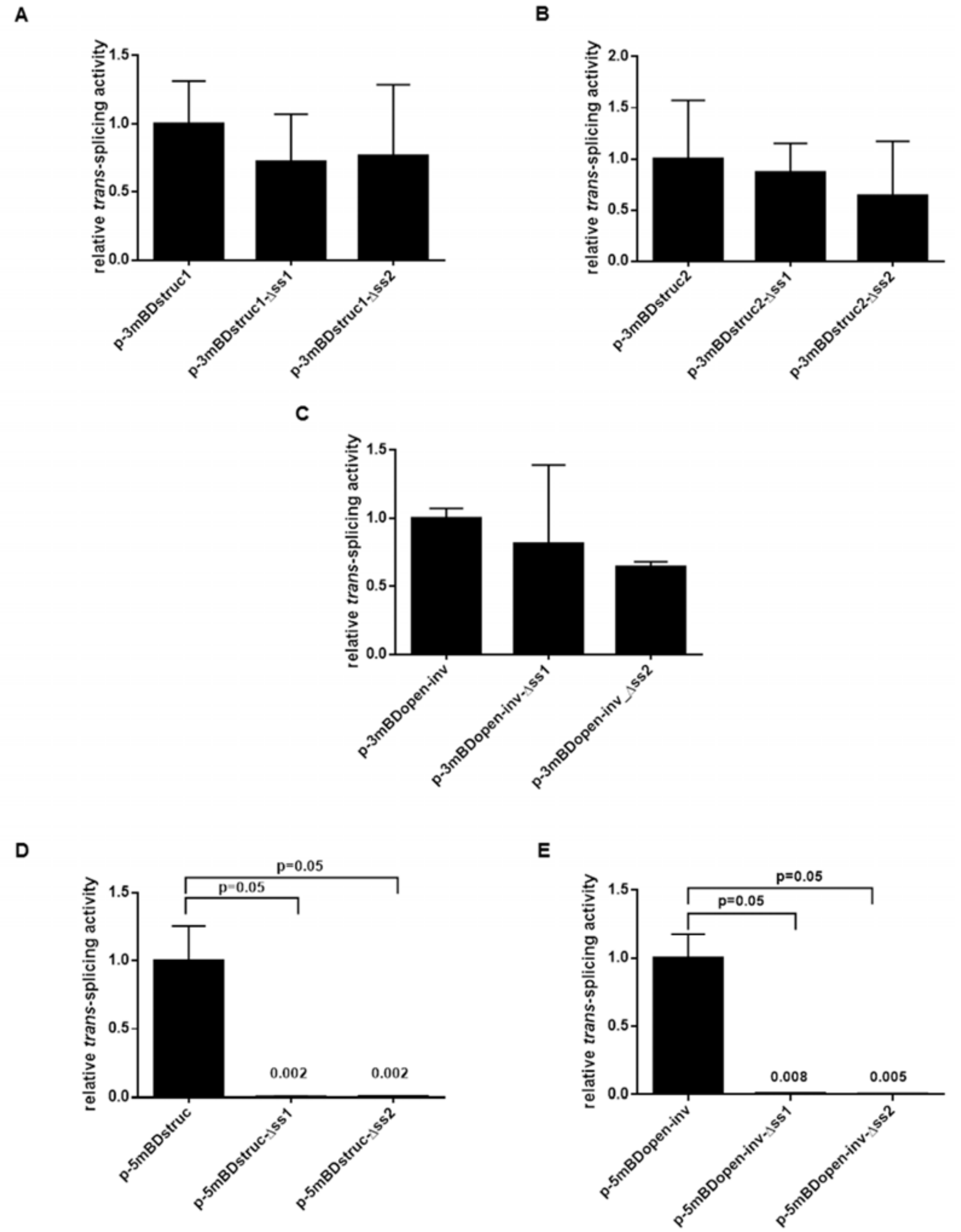

Figure S5. Relative *trans*-splicing activities of 3' and 5'ER tsRNAs harboring structured BDs (Referring to Figure 2). Comparison of constructs with consensus splice sites and splice site mutations. Mean ± SEM (n=3). Significance was tested using One-way ANOVA with Tukey post-hoc test.

A **p-5mBDopen**

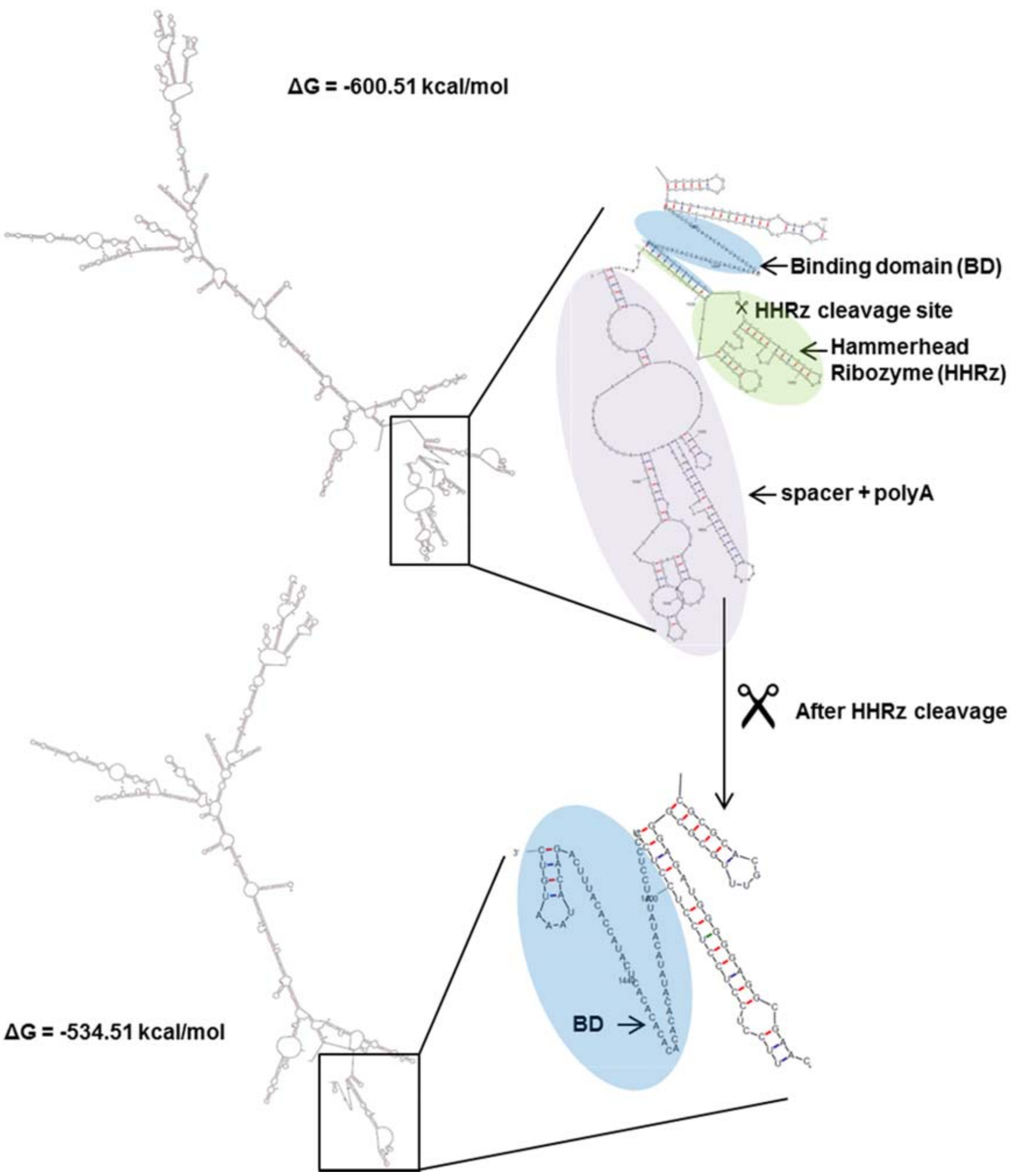
ΔG = -600.51 kcal/mol
Binding domain (BD)
HHRz cleavage site
Hammerhead Ribozyme (HHRz)
spacer + polyA
After HHRz cleavage
BD
ΔG = -534.51 kcal/mol

B p-5mBDopen-hp

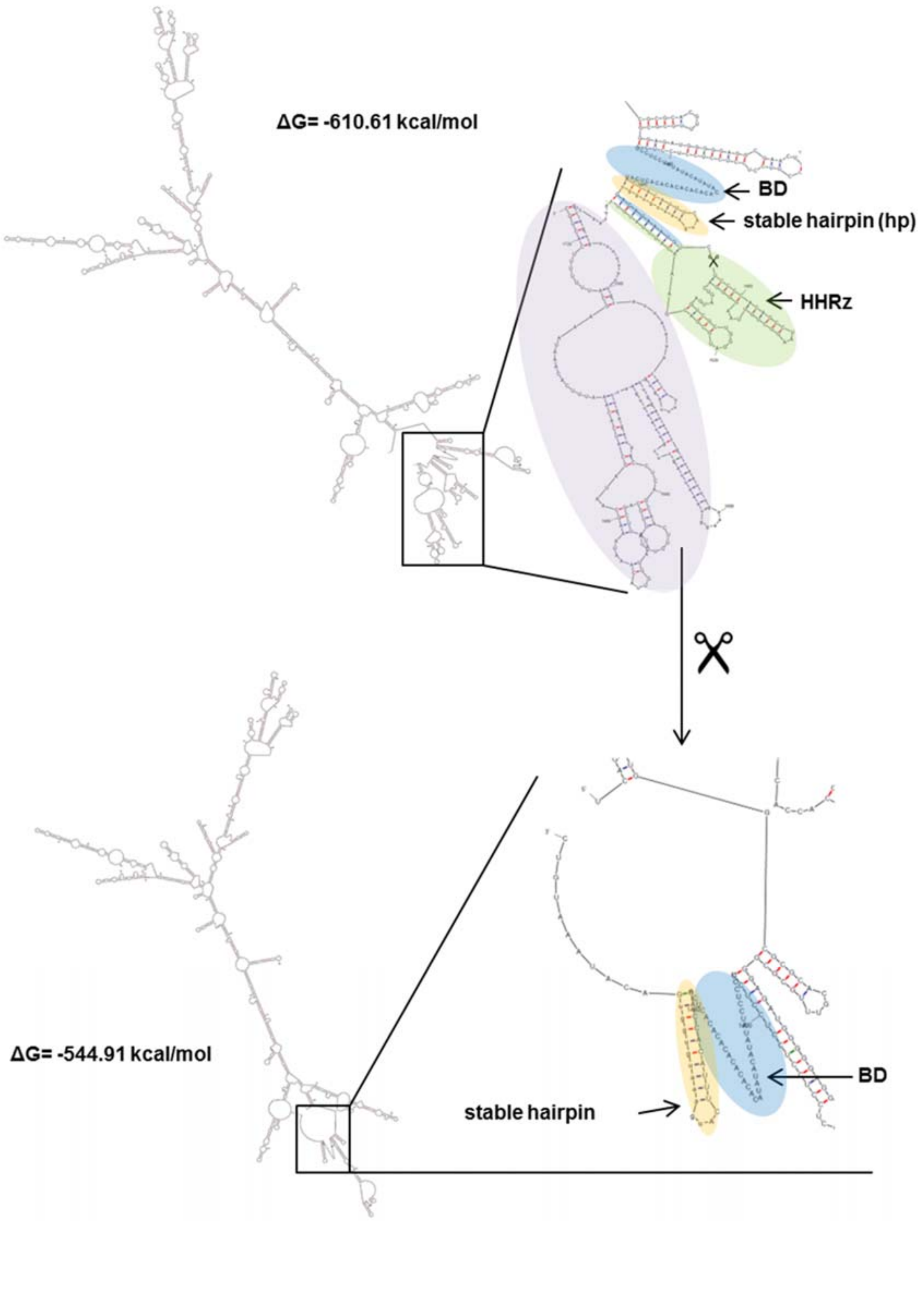

c **p-5mBDopen-Y**

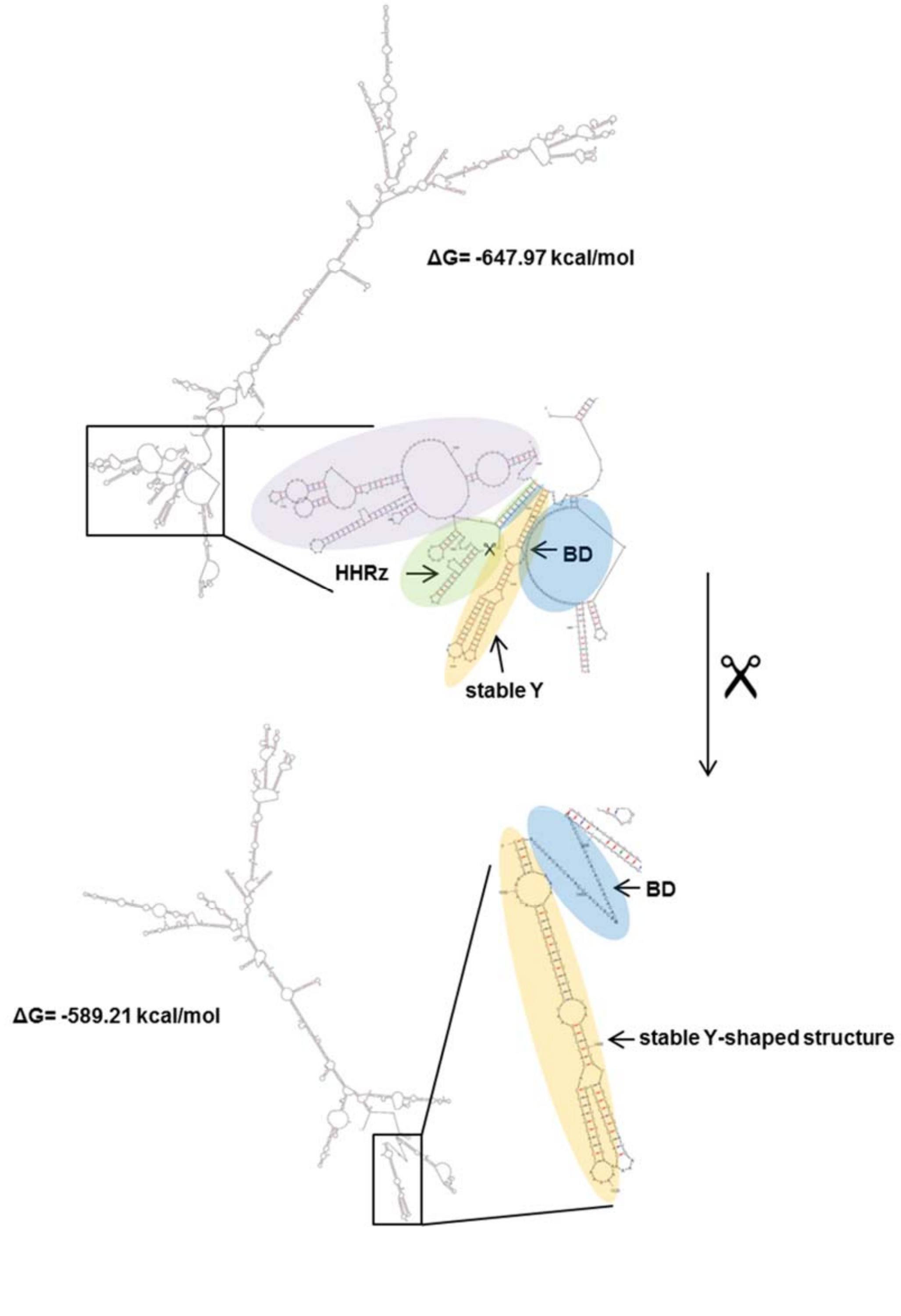

D p-5mBDopen-ΔHH

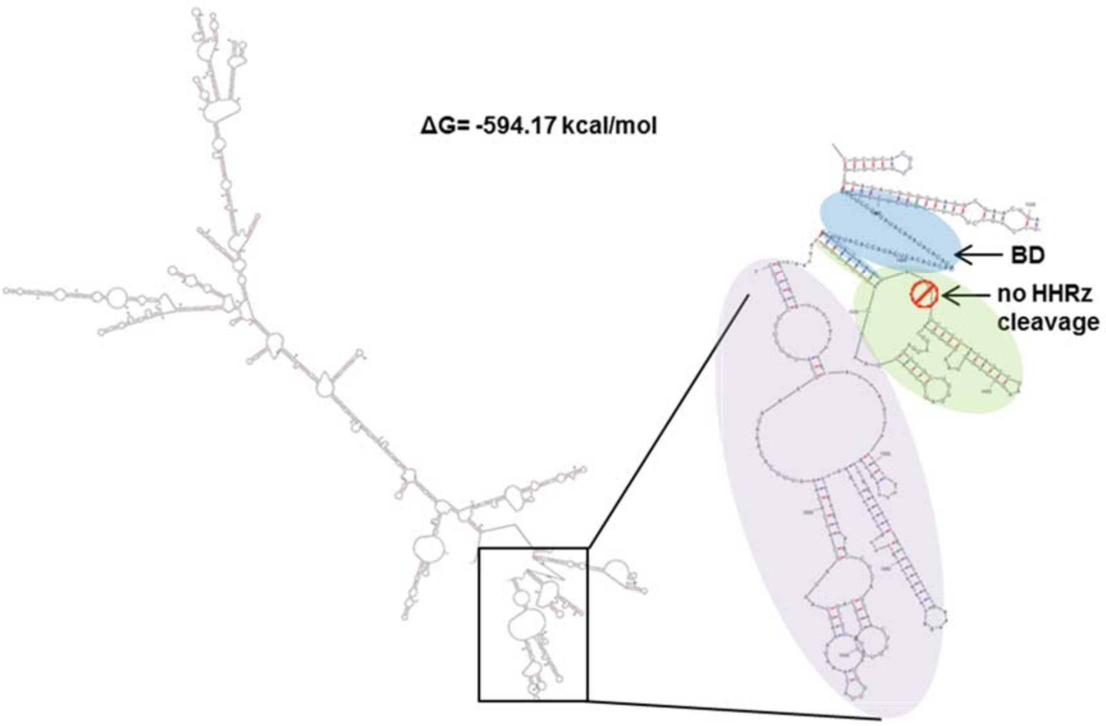

E p-5mBDopen-HH(-)

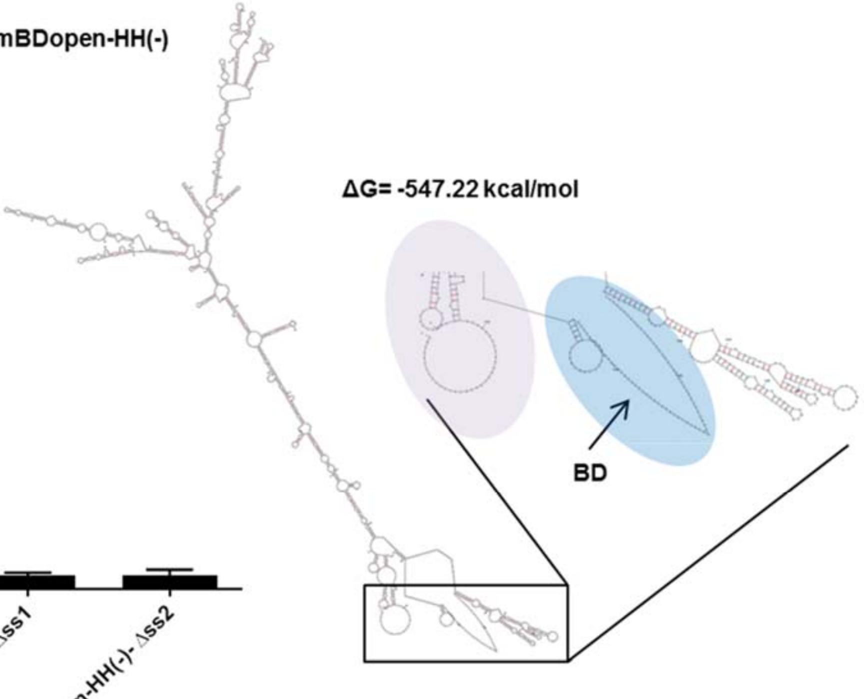

F

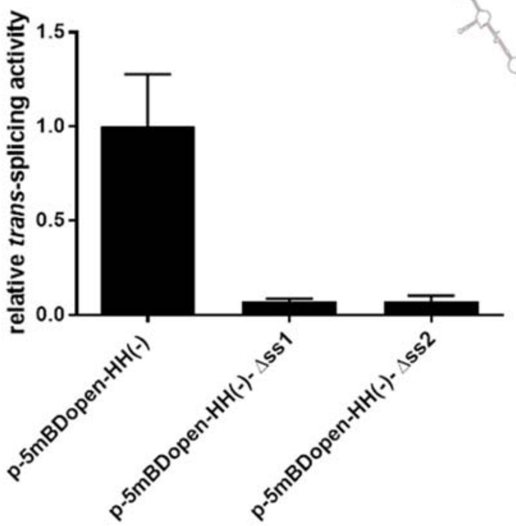

Figure S6. RNA secondary structure predictions of 5'ER tsRNA with 3' end modifications (Referring to Figure 3). (**A**) Secondary structure of parental construct p-5mBDopen. Upper panel: before HHRz cleavage with the open BD, followed by the HHRz with the active cleavage site, a spacer that facilitates correct folding of the HHRz, and a polyA site; lower panel: after HHRz cleavage with the 3' terminal open BD but without HHRz, spacer, and polyA site. (**B**) Secondary structure of construct p-5mBDopen-hp. Upper panel: before HHRz cleavage with the open BD, followed by a hairpin-loop structure (hp), the HHRz with the active cleavage site, a spacer that facilitates correct folding of the HHRz, and a polyA site; lower panel: after HHRz cleavage with the open BD followed by a 3' terminal hp structure but without HHRz, spacer, and polyA site. (**C**) Secondary structure of construct p-5mBDopen-Y. Upper panel: before HHRz cleavage with the open BD, followed by a Y-shaped structure (Y), the HHRz with the active cleavage site, a spacer that facilitates correct folding of the HHRz, and a polyA site; lower panel: after HHRz cleavage with the open BD followed by a 3' terminal Y-shaped structure but without HHRz, spacer, and polyA site. (**D**) Secondary structure of construct p-5mBDopen-DHH with the open BD, followed by the HHRz with the inactive cleavage site, a spacer that facilitates correct folding of the HHRz, and a polyA site. (**E**) Secondary structure of construct p-5mBDopen-HH(-) with the open BD followed by a polyA site. This construct doesn't have a HHRz or spacer. (A-E) BD (blue shading); 3' end stabilizing domain (orange shading); HHRz (green shading); polyA with or without spacer (purple shading). (**F**) Relative *trans*-splicing activity of construct p-5mBDopen-HH(-) compared with its ss mutants. Mean ± SEM (n=3). Significance was tested using One-way ANOVA with Tukey post-hoc test.

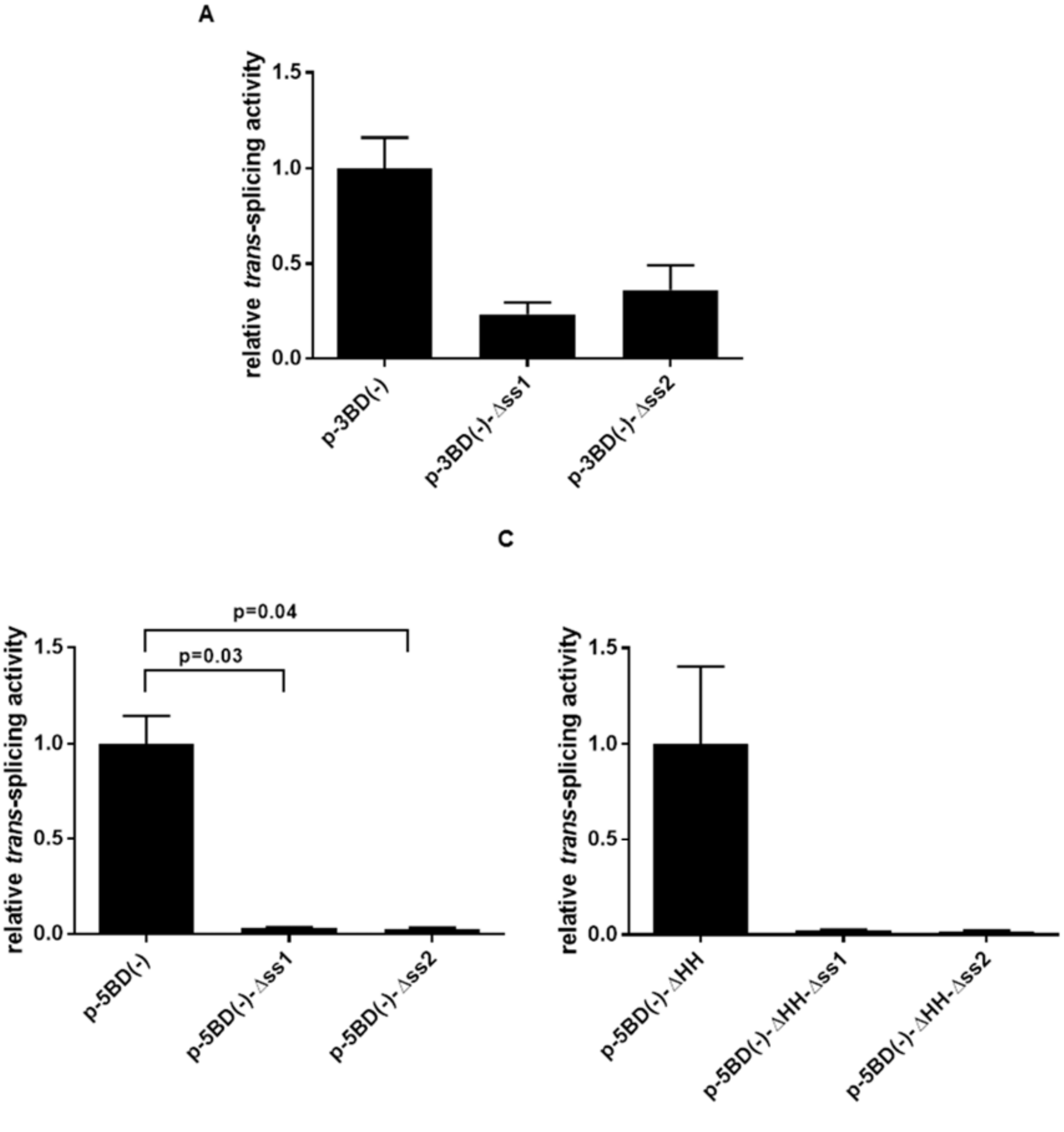

Figure S7. Relative *trans*-splicing activities of BD-negative constructs and corresponding splice site mutants (Referring to Figure 5). (**A**) 3'ER construct p-3BD(-) and ss mutants. (**B**) 5'ER construct p-5BD(-) and ss mutants. (**C**) 5'ER construct p-5BD(-)-ΔHH and ss mutants. Mean ± SEM (n=1-3), Significance was tested using One-way ANOVA with Tukey post-hoc test.

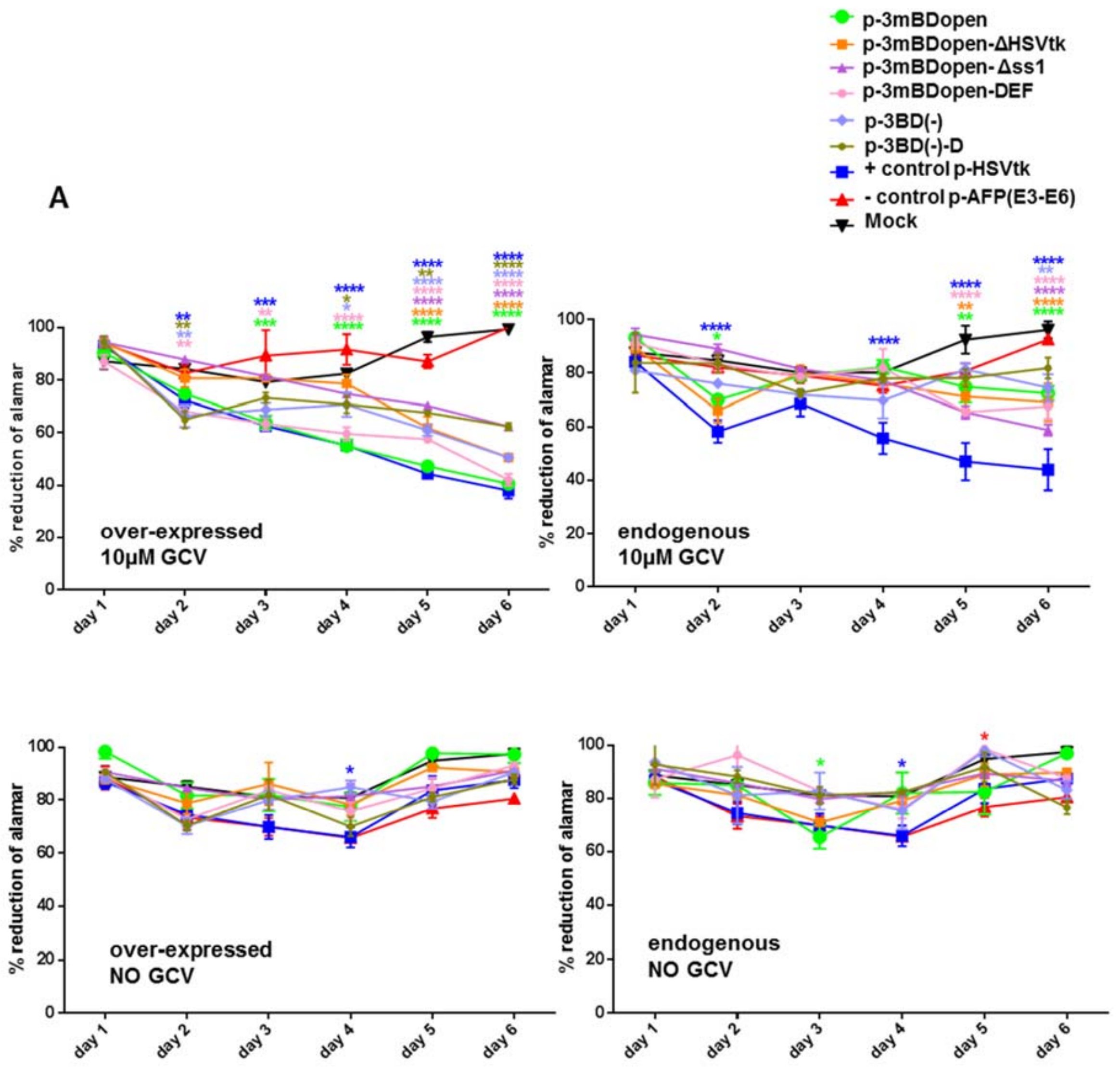
A
p-3mBDopen
p-3mBDopen-ΔHSVtk
p-3mBDopen-Δss1
p-3mBDopen-DEF
p-3BD(-)
p-3BD(-)-D
+ control p-HSVtk
- control p-AFP(E3-E6)
Mock
% reduction of alamar
over-expressed
10μM GCV
endogenous
10μM GCV
over-expressed
NO GCV
endogenous
NO GCV
day 1
day 2
day 3
day 4
day 5
day 6

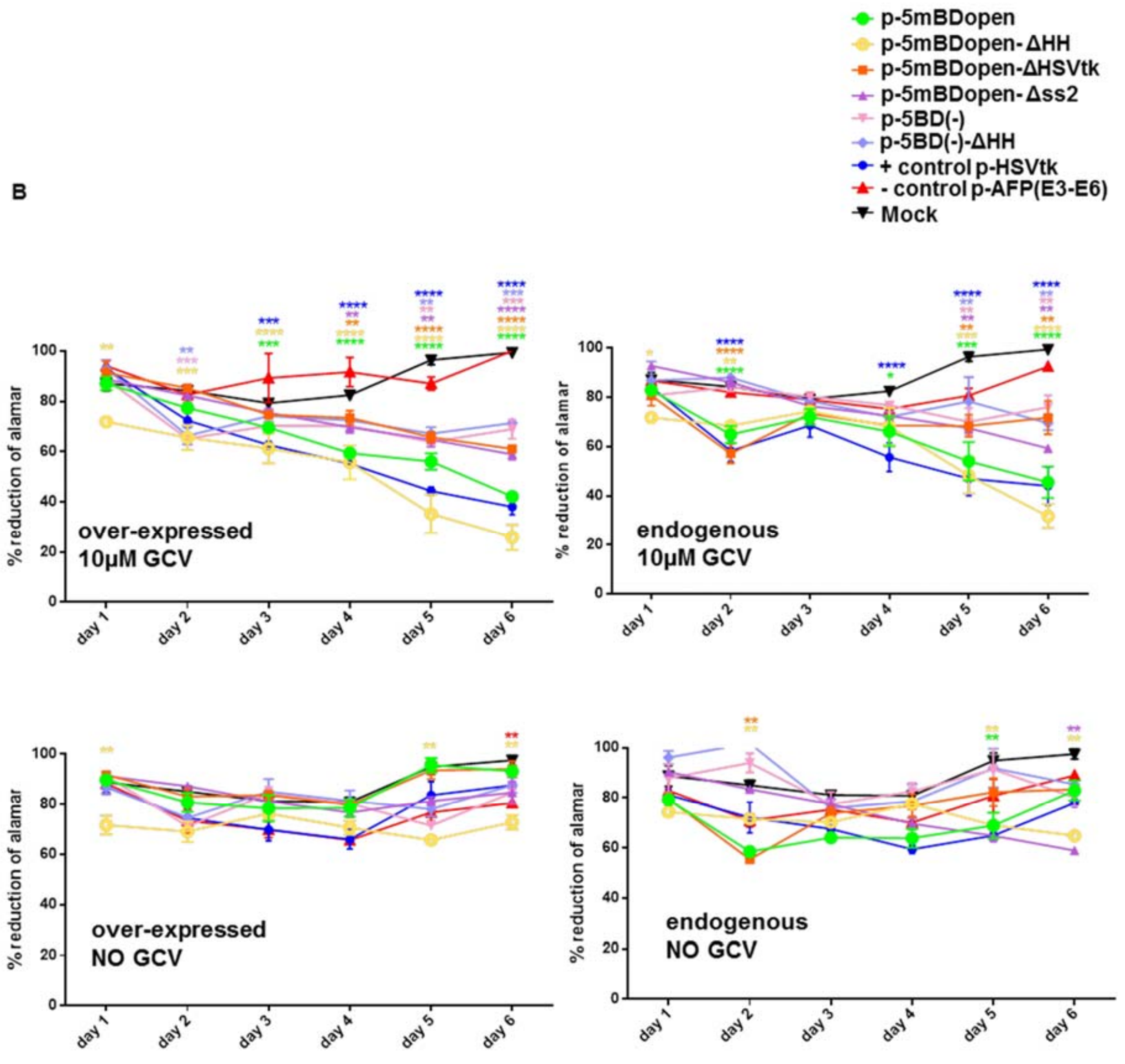

Figure S8. Death of AFP expressing HepG2 cells triggered by *trans*-splicing toward the over-expressed or endogenous AFP message (Referring to Figure 6). Alamar Blue cell viability assay with (**A**) 3'ER or (**B**) 5'ER constructs using cells with over-expressed (upper left panel) or endogenous AFP levels (upper right panel) and 10 µM GCV over a period of 6 days. Lower left or right panels show no drug control assays using cells with over-expressed or endogenous AFP levels. The 5'ER construct with the mutated HHRz cleavage site 5mBDopen-ΔHH represents a fully intact mRNA harbouring a 5' cap and a 3' poly-A tail even without undergoing *trans*-splicing. Hence, the high cell death activity of this tsRNA might be either due to *trans*-splicing independent nuclear export and HSVtk translation and/or alternatively due to high endogenous stability and subsequent high rates of *trans*-splicing which indeed was measured for this RNA (Fig. 3D). Mean ± SEM (n=3). Significance calculated with respect to mock. Significance relative to mock was tested using Two-way ANOVA with Bonferroni post-hoc test. * $p<0.05$, ** $p<0.01$, *** $p<0.001$ and **** $p<0.0001$.

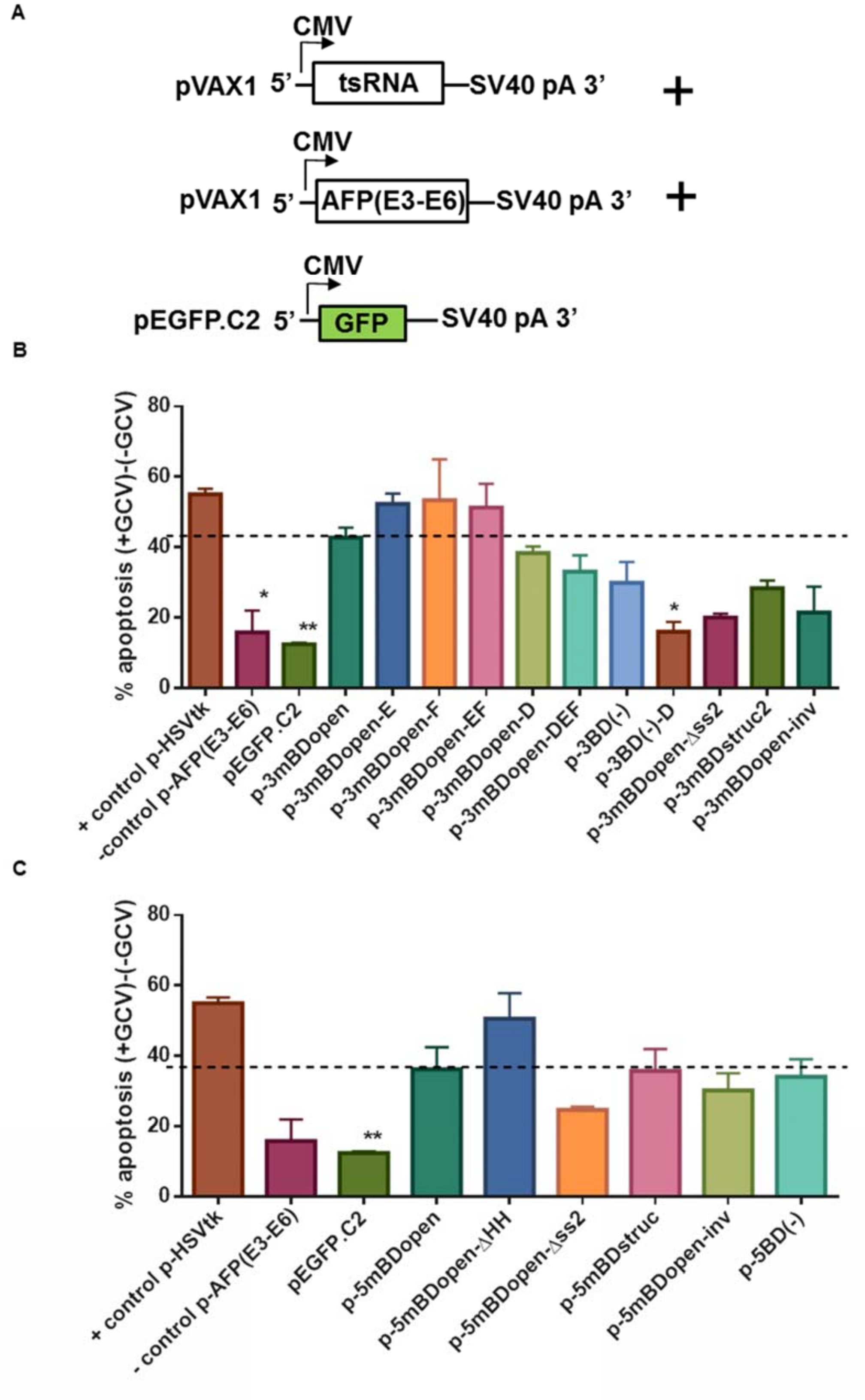
A
CMV
pVAX1 5' tsRNA SV40 pA 3' +
CMV
pVAX1 5' AFP(E3-E6) SV40 pA 3' +
CMV
pEGFP.C2 5' GFP SV40 pA 3'
B
% apoptosis (+GCV)-(-GCV)
80
60
40
20
0
*
**
*
+ control p-HSVtk
-control p-AFP(E3-E6)
pEGFP.C2
p-3mBDopen
p-3mBDopen-E
p-3mBDopen-F
p-3mBDopen-EF
p-3mBDopen-D
p-3mBDopen-DEF
p-3BD(-)
p-3BD(-)-D
p-3mBDopen-Δss2
p-3mBDstruc2
p-3mBDopen-inv
C
% apoptosis (+GCV)-(-GCV)
80
60
40
20
0
**
+ control p-HSVtk
- control p-AFP(E3-E6)
pEGFP.C2
p-5mBDopen
p-5mBDopen-ΔHH
p-5mBDopen-Δss2
p-5mBDstruc
p-5mBDopen-inv
p-5BD(-)

D

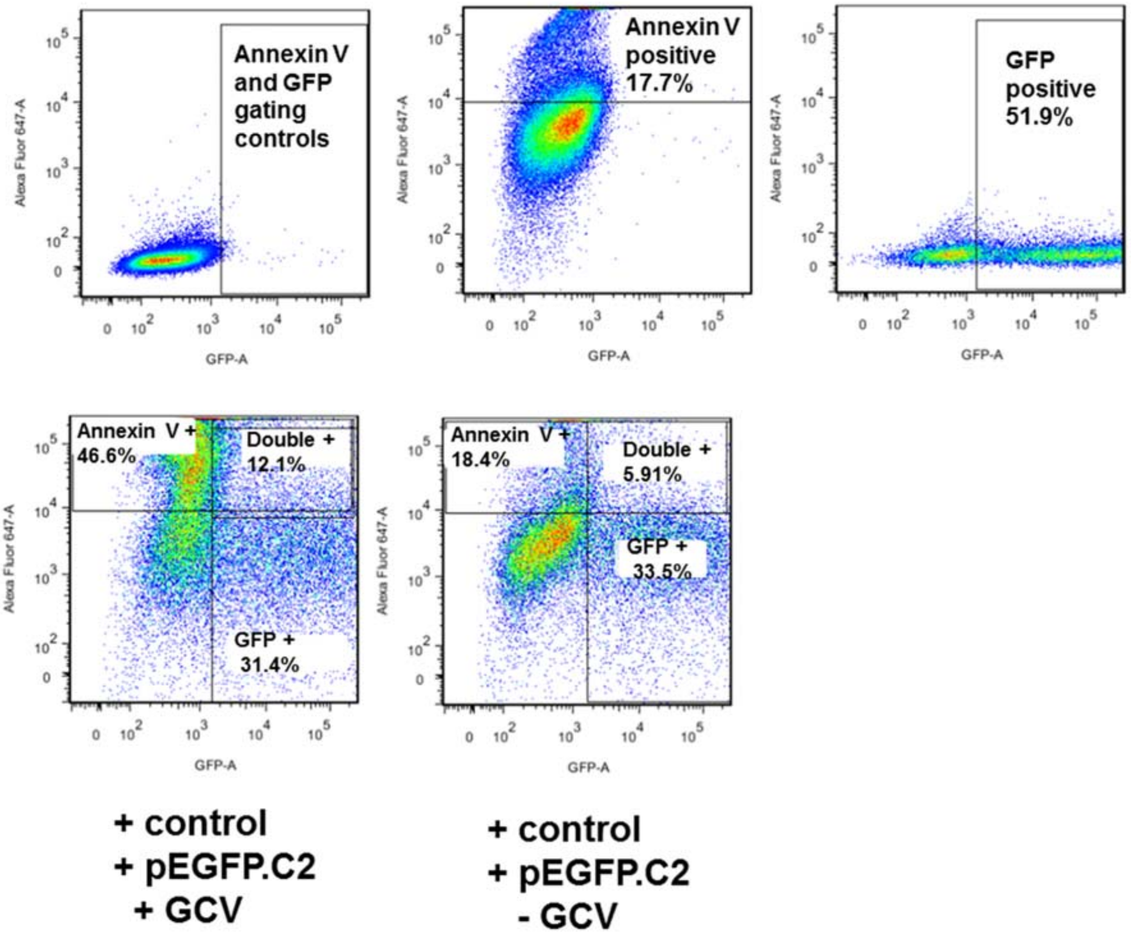

Figure S9. Annexin V/PI apoptosis assay of HepG2 cells co-transfected with *trans*-splicing vectors, AFP mini-gene, and pEGFP (Referring to Figure 6). (**A**) Design of vectors used for the Annexin V/PI apoptosis assay: *trans*-splicing vectors (pVAX1 backbone), AFP mini-gene vector (pVAX1 backbone), and EGFP expression vector pEGFP.C2. HepG2 cells were co-transfected including vectors for 3’ER (**B**) or 5’ER (**C**) and 24 hours post transfection treated with 100 µM GCV for 48 hours. The empty pSUPER plasmid was used as feeder DNA to top-up the total amount of transfected DNA in the negative (AFP mini-gene or EGFP) or positive (p-HSVtk) controls. (B, C) Mean ± SEM (n=3). Significance relative to the parental constructs p-3mBDopen (B) or p-5mBDopen (C) was tested using Two-way ANOVA with Tukey post-hoc test. * $p<0.05$, ** $p<0.01$. (**D**) Representative 2-D scatter plots of flow cytometry data showing the gating strategy to distinguish Annexin V-positive apoptotic cells, EGFP-positive cells, and double-positive cells. The example shows cells transfected with the p-HSVtk positive control with (+) or without (-) 100 µM GCV treatment. The relatively large populations of single-positive (either Annexin V or EGFP) cells indicate sub-optimal co-transfection efficiencies.

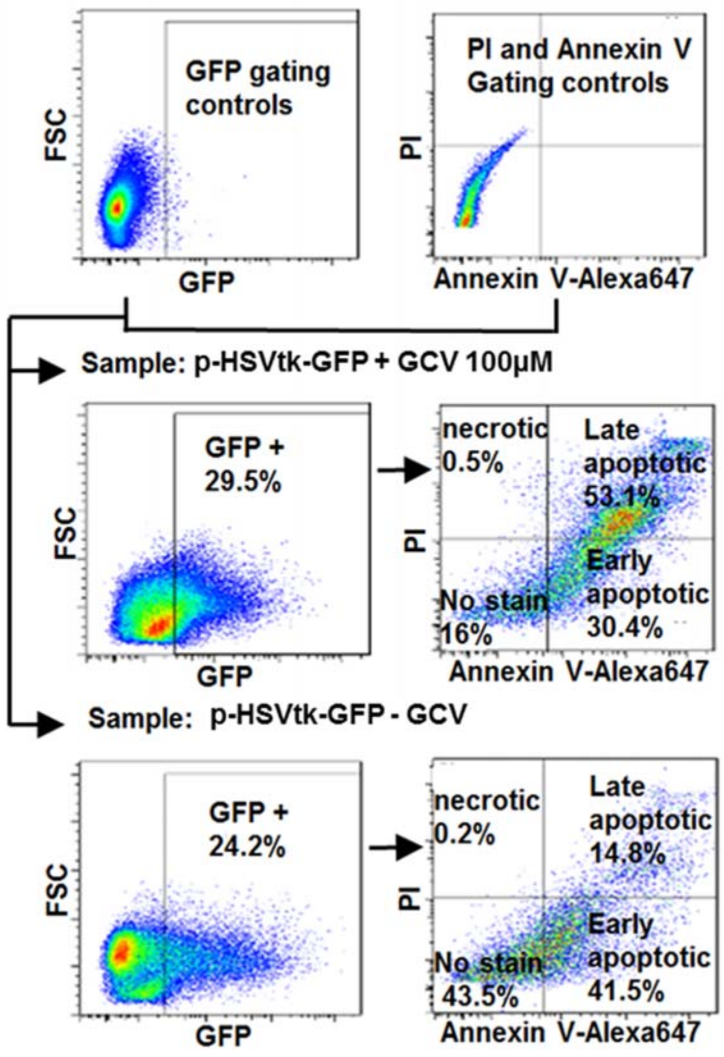

Figure S10. Gating strategy for the Annexin V/PI apoptosis assays of HepG2 cells transfected with EGFP-expressing *trans*-splicing vectors (Referring to Figure 10). Representative 2-D scatter plots of flow cytometry data allow to distinguish between *trans*-splicing-triggered early and late apoptosis. The example shows cells transfected with the p-HSVtk-GFP positive control with (+) or without (-) 100 µM GCV treatment.

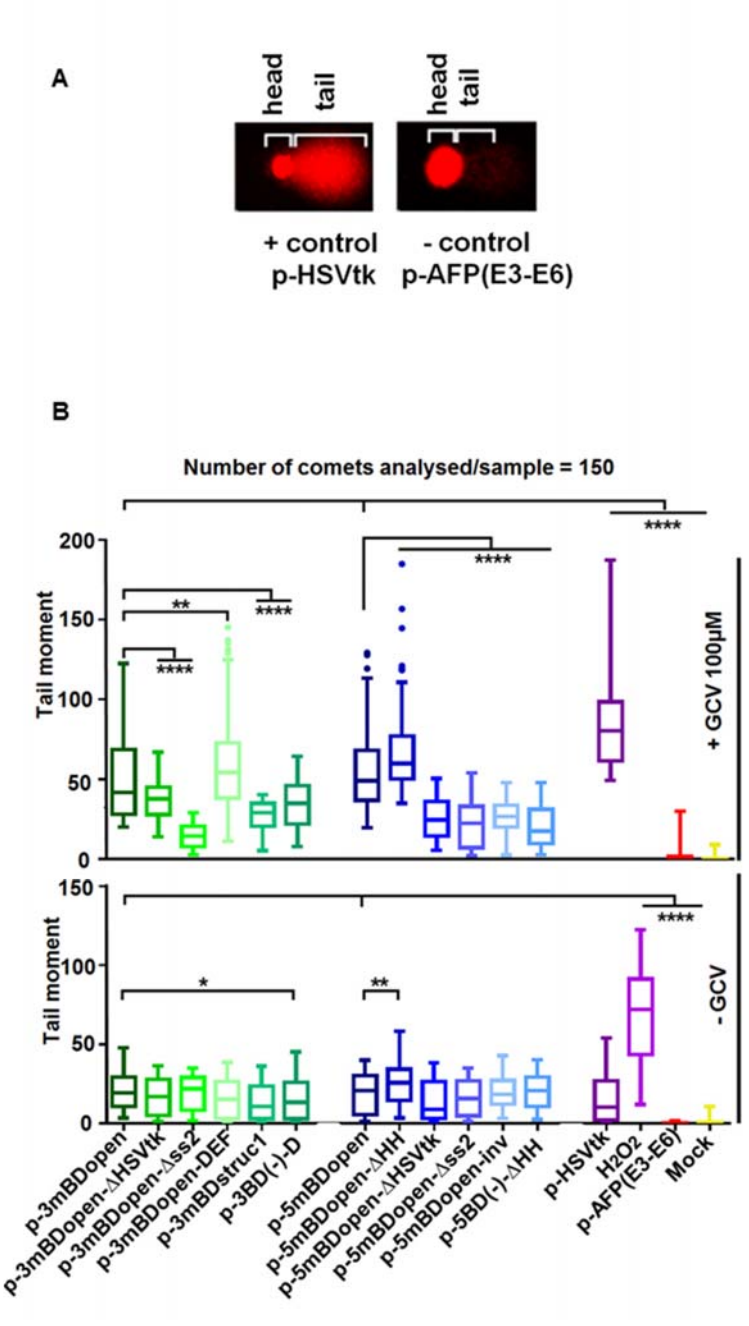

Figure S11. Comet assay in HepG2 cells co-transfected with *trans*-splicing vectors and AFP mini-gene ((Referring to Figure 11). (**A**) Representative images of the comet assay with the position of head and tail taken at 10x magnification using a confocal microscope. (**B**) Detection of cell death triggered by tsRNA using the comet assay and reflected by the tail moment caused by damaged DNA 24 hours post 100 µM GCV treatment (upper panel) or in the absence of GCV (lower panel). 150 comets were analysed for each sample. Box plots indicate mean ± SEM (n=3). Tail moments above Q3 + 1.5 (IQR) are shown as dots (outliers). Cells treated with hydrogen peroxide were used as positive control. The negative controls including the no GCV control didn't show significant DNA damage. Significance was tested using One-way ANOVA with Tukey post-hoc test. * $p<0.05$, ** $p<0.01$ and **** $p<0.0001$.

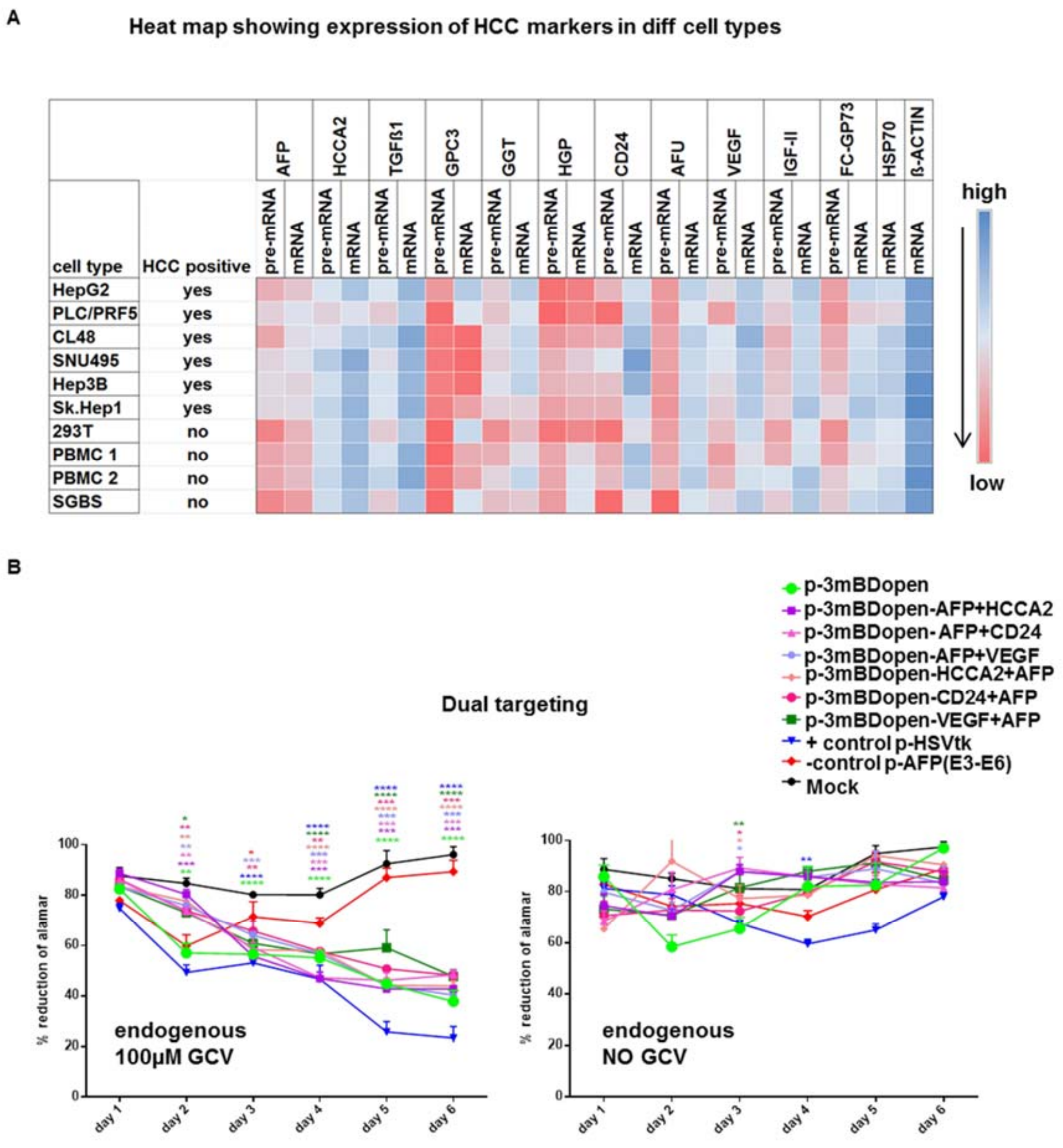

Figure S12. Cell death triggered by *trans*-splicing of dual-targeting tsRNAs toward two endogenous pre-mRNA targets (Referring to Figure 7). (**A**) Heat map showing pre-mRNA and mRNA expression levels of HCC biomarker genes in various cell types including HCC cell lines as well as HCC-unrelated cell lines, peripheral blood mononuclear cells (PBMCs), and primary adipocytes. Based on abundance, HCC-specificity, and clinical significance, three secondary targets were selected: HCCA2 (YY1AP1) is a HCC-associated protein that is upregulated in liver cancer patients.[1] CD24 represents a surface marker glycoprotein and marker for high invasiveness and metastatic potential of cells in late stage HCC.[2,3] VEGF is a cytokine playing an important role in tumour angiogenesis and is a biomarker of lymph node metastasis in HCC.[4] (**B**) Alamar Blue cell viability assay comparing the levels of death triggered by dual-targeting and the parental AFP-only targeting 3'ER constructs in cells expressing endogenous AFP levels after 100 µM GCV treatment (left) or no drug treatment (right). Mean ± SEM (n=3). Significance was tested using Two-way ANOVA with Bonferroni post-hoc test compared to mock. * $p<0.05$, ** $p<0.01$, *** $p<0.001$ and **** $p<0.0001$.

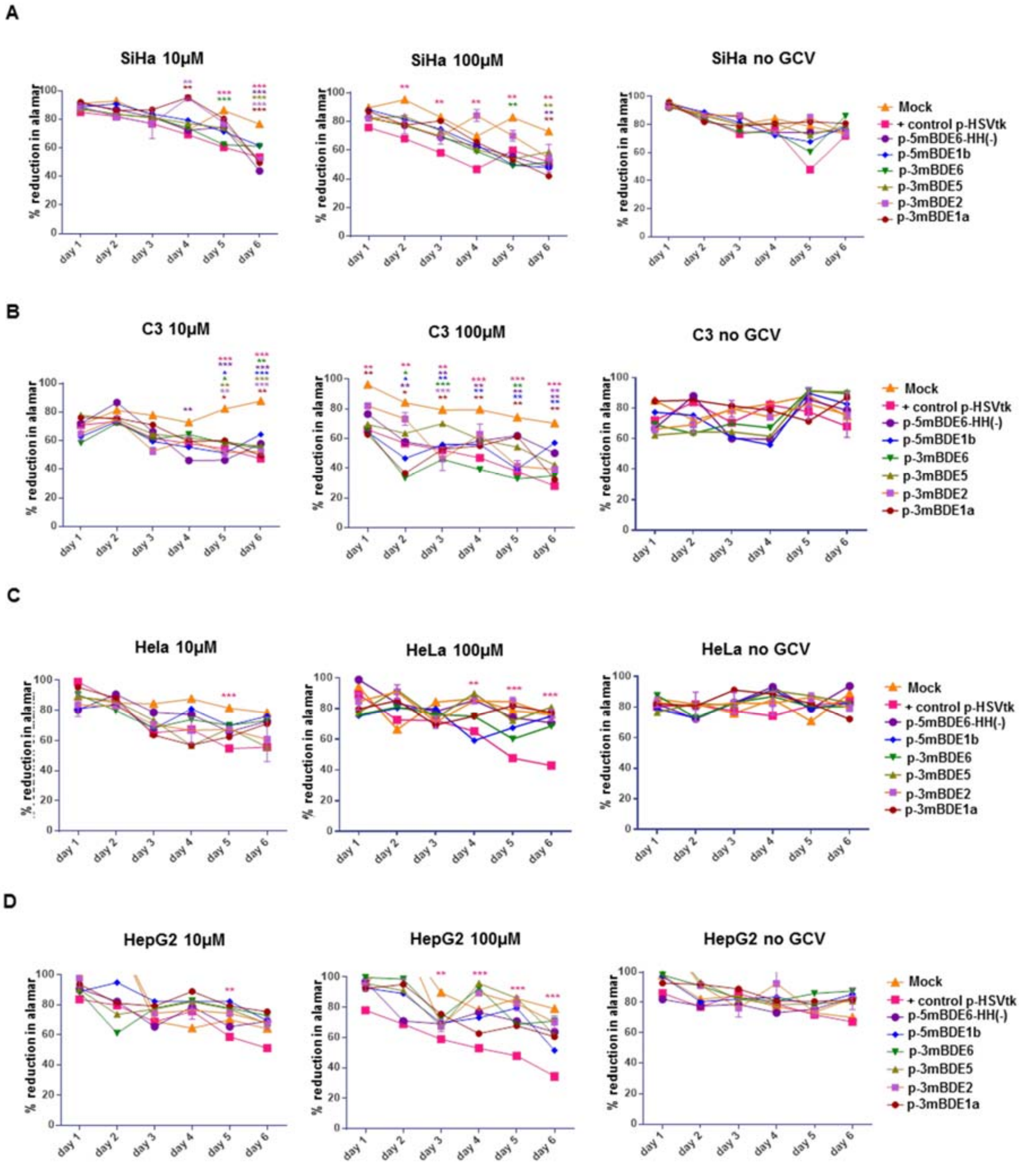

Figure S13. Cell death triggered by HPV-16-targeting *trans*-splicing vectors at 10 µM, 100 µM or no GCV treatment (Referring to Figure 8). Two 5'ER tsRNA constructs targeting E1b and E6 and four 3'ER tsRNA constructs targeting E1a, E2, E5 and E6 were tested. (**A**) HPV-16-positive human cell line SiHa; (**B**) HPV-16-positive mouse cell line C3; (**C**) HPV-18-positive human cell line HeLa; and (**D**) HPV-negative human cell line HepG2. Cells were treated with the indicated GCV concentrations for 6 consecutive days. Mean ± SEM (n=3). Significance was tested using Two-way ANOVA with Bonferroni post-hoc test compared to mock. * p<0.05, ** p<0.01, *** p<0.001 and **** p<0.0001.

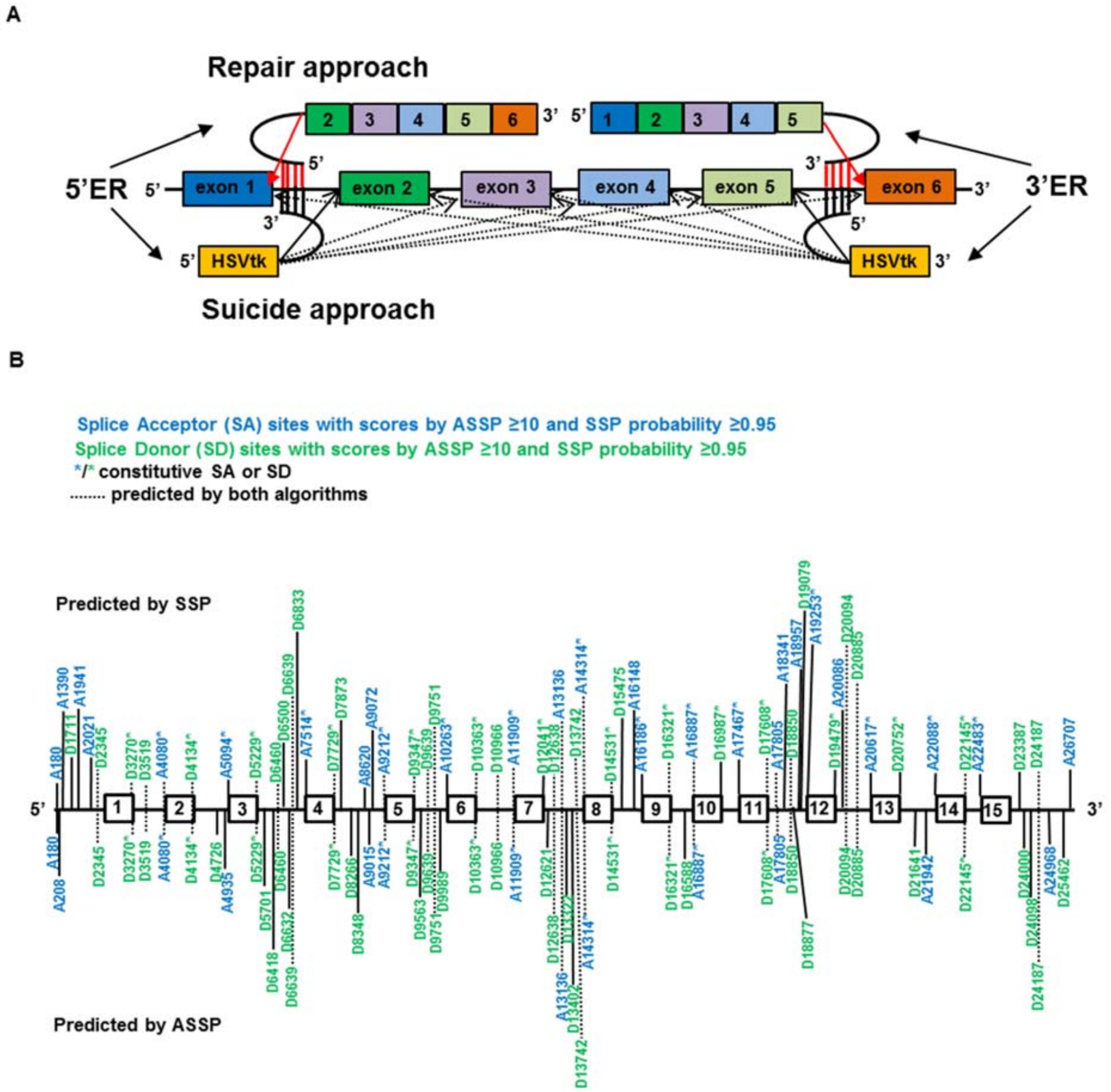

Figure S14. Alternative splice site targeting (Referring to Figure 4). (**A**) Conceptual differences in *trans*-splicing based repair and suicide gene therapy. The cartoon depicts a target gene composed of six exons and five introns. In suicide gene therapy, 5'/3'ER tsRNAs are recommended to target the very first/last intron in order to enable *trans*-splicing toward the multiple alternative SA/SD sites. In repair gene therapy, only those 5'/3'ER tsRNAs targeting the very last/first intron are not impaired by alt-on-ts. Hence in order to avoid alt-on-ts, a repair tsRNA for 5'/3'ER would need to provide all but the very last/first target exons. (**B**) Pattern of SA and SD sites on AFP pre-mRNA predicted using the ASSP and SSP software tools.

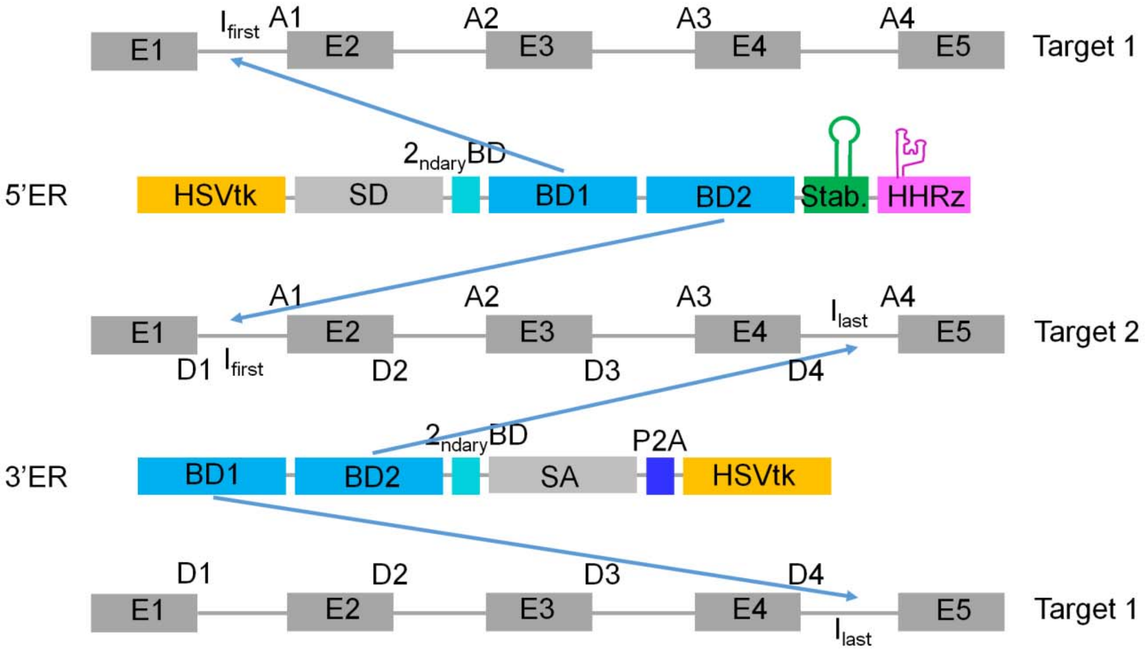

Figure S15. Schematic drawing highlighting recommendations for the rational design of *trans*-splicing based suicide vectors. The cartoon depicts the recommended design of 5' and 3'ER tsRNAs for a HSVtk/GCV suicide gene therapy approach. Exemplary target pre-mRNAs target 1 and target 2 are composed of five exons (E1 to E5) and four introns. Beyond standard design features, the recommended rational design comprises the use of one or more unstructured primary BDs (blue) for efficient targeting of one or more pre-mRNA biomarkers which are specific for the targeted cell type. The given example shows dual-targeting tsRNAs for 5' or 3'ER harboring two BDs (BD1 and BD2) for targeting two target messages. Provided unstructured BDs can be identified, then tsRNAs for 5' or 3'ER are recommended to target the very first (I_{first}) or last (I_{last}) intron of the respective targets in order to enable *trans*-splicing toward any of the target SA or SD sites for maximum on-target activity. A secondary BD (cyan) shielding the SD (5'ER) or SA (3'ER) sites of the tsRNA in the absence of target binding may be considered to suppress off-target *trans*-splicing. BDs of tsRNA for 5'ER should be followed downstream by a thermodynamically stable RNA secondary structure domain (Stab.) and ribozyme that cleaves of the polyA tail for nuclear targeting.

B, Supplemental Tables

No.	CONSTRUCTS		ADDITIONAL FEATURES OR MODIFICATIONS									
		HSVtk mutant	Splice site mutant	Inactive HHRz Cleavage	Structured BD	Fully complemen-tary to target BD	No BD	No HHRz	Stable secondary structure at 3'end	Additional BDs	Double BDs	+ GFP cassette
**3' *Trans*-splicing plasmids targeting AFP**												
1	p-3mBDopen											
2	p-3mBDopen-ΔHSVtk	√										
3	p-3mBDopen-Δss1		√									
4	p-3mBDopen-Δss2		√									
5	p-3mBDstruc1				√							
6	p-3mBDstruc2				√							
7	p-3mBDopen-inv				√							
8	p-3mBDstruc1-Δss1		√		√							
9	p-3mBDstruc2-Δss1		√		√							
10	p-3mBDopen-inv-Δss1		√		√							
11	p-3mBDstruc1-Δss2		√		√							
12	p-3mBDstruc2-Δss2		√		√							
13	p-3mBDopen-inv-Δss2		√		√							
14	p-3cBDopen					√						
15	p-3cBDopen-Δss1		√			√						
16	p-3cBDopen-Δss2		√			√						
17	p-3cBDstruc1				√	√						
18	p-3cBDstruc2				√	√						
19	p-3cBDopen-inv				√	√						
20	p-3BD(-)						√					
21	p-3BD(-)-Δss1		√				√					
22	p-3BD(-)-Δss2		√				√					
23	p-3mBDopen-E									√		
24	p-3mBDopen-F									√		
25	p-3mBDopen-EF									√		
26	p-3mBDopen-D									√		
27	p-3mBDopen-DEF									√		
28	p-3BD(-)-D						√			√		
29	p-3mBDopen-GFP											√
30	p-3mBDopen-DEF-GFP									√		√
31	p-3mBDopen-ΔHSVtk-GFP	√										√
32	p-3mBDopen-Δss1-GFP		√									√
**5' *Trans*-splicing plasmids targeting AFP**												
33	p-5mBDopen											
34	p-5mBDopen-ΔHSVtk	√										
35	p-5mBDopen-Δss1		√									
36	p-5mBDopen-Δss2		√									
37	p-5mBDopen-ΔHH			√								
38	p-5mBDstruc				√							
39	p-5mBDopen-inv				√							
40	p-5mBDstruc-Δss1		√		√							
41	p-5mBDopen-inv-Δss1		√		√							
42	p-5mBDstruc-Δss2		√		√							
43	p-5mBDopen-inv-Δss2		√		√							
44	p-5cBDopen					√						
45	p-5cBDopen-Δss1		√			√						
46	p-5cBDopen-Δss2		√			√						
47	p-5cBDstruc				√	√						
48	p-5cBDopen-inv				√	√						
49	p-5mBDopen-hp								√			
50	p-5mBDopen-Y								√			
51	p-5BD(-)						√					
52	p-5BD(-)-ΔHH			√			√					

No.	CONSTRUCTS		ADDITIONAL FEATURES OR MODIFICATIONS									
		**HSVtk mutant**	**Splice site mutant**	**Inactive HHRz Cleavage**	**Structured BD**	**Fully complementary to target BD**	**No BD**	**No HHRz**	**Stable secondary structure at 3'end**	**Additional BDs**	**Double BDs**	**+ GFP cassette**
53	p-5BD(-)-Δss1		√				√					
54	p-5BD(-)-Δss2		√				√					
55	p-5BD(-)-ΔHH-Δss1		√	√			√					
56	p-5BD(-)-ΔHH-Δss2		√	√			√					
57	p-5mBDopen-HH(-)							√				
58	p-5mBDopen-HH(-)-Δss1		√					√				
59	p-5mBDopen-HH(-)-Δss2		√					√				
60	p-5mBDopen-GFP											√
61	p-5mBDopen-ΔHH-GFP			√								√
62	p-5mBDopen-ΔHSVtk-GFP	√										√
63	p-5mBDopen-Δss2-GFP		√									√
**3' *Trans*-splicing plasmids dual targeting HCC markers**												
64	p-3mBDopen-AFP+HCCA2										√	
65	p-3mBDopen-AFP+CD24										√	
66	p-3mBDopen-AFP+VEGF										√	
67	p-3mBDopen-HCCA2+AFP										√	
68	p-3mBDopen-CD24+AFP										√	
69	p-3mBDopen-VEGF+AFP										√	
***Trans*-splicing plasmids targeting HPV-16**												
70	p-3mBDE1a											
71	p-3mBDE2											
72	p-3mBDE5											
73	p-3mBDE6											
74	p-5mBDE1b											
75	p-5mBDE6-HH(-)							√				
**Control plasmids**												
76	p-HSVtk											
77	p-AFP(E3-E6)											
78	p-HSVtk-GFP											√
79	p-AFP(E3-E6)-GFP											√
80	p-GL3-GFP											√

Table S1. List of Constructs.

Target	tsRNA	Potential BDs<br>5'-target-3'<br>3'-tsRNA-5'			
		Position on target	Length	Sequence	Probability
AFP E3-E6	p-3BD(-)	+ 75 (intron 3)	11mer	5′-GCAGUGAAAAA-3′<br>3′-CGUCACUUUUU-5′	0.68
		+ 355 (intron 5)	11mer	5′-AAAUGCUUUAU-3′<br>3′-UUUACGAAAUA-5′	
AFP E3-E6	p-5BD(-) and p-5BD(-)-ΔHH	+ 75 (intron 3)	11mer	5′-GCAGUGAAAAA-3′<br>3′-CGUCACUUUUU-5′	0.75
		+ 355 (intron 5)	11mer	5′-AAAUGCUUUAU-3′<br>3′-UUUACGAAAUA-5′	

Table S2. Accidental complementarity between BD-negative tsRNA and the AFP mini-gene message. (Referring to Figure 4)

Table S3. List of oligonucleotides, probes, and primers. (Separate file)

C, Supplemental Materials and Methods

Plasmid construction. The AFP mini-gene p-AFP(E3-E6) consisting of exons 3-6 and introns 3 and 5 (NCBI Nucleotide: M16110) was generated by gene synthesis (GeneArt, Regensburg) and cloned into pVAX1 using *Nhe*I and *Kpn*I. The HSVtk positive control was generated by sub-cloning the 1136 bp HSVtk gene from the parental *trans*-splicing vector into pVAX using *Sac*I and *Bam*HI. Modified sequences were generated either by gene synthesis or PCR mutagenesis and sub-cloned into the parental vectors p-3mBDopen or p-5mBDopen. Mutated 3' splice site (ss) were sub-cloned into p-3mBDopen using *Bbv*CI and *Sac*I or *Nhe*I and *Pvu*I generating constructs p-3mBDopen-Δss1 or p-3mBDopen-Δss2 by replacing the wild-type (wt) ss. Construct p-5mBDopen-Δss1 was generated by replacing the wt-SD of p-5mBDopen by the mutated SD (Δss1) using *Bss*HII and *Bbs*I. To generate construct p-5mBDopen-Δss2, the mutated SD (Δss2) was synthesised using nested PCR and cloned into the parental vector using *Bss*HII and *Kpn*I. To generate the inactive HSVtk mutants p-3mBDopen-ΔHSVtk and p-5mBDopen-ΔHSVtk, the ΔHSVtk gene was cloned into the parental vectors using *Pvu*I and *Pst*I or *Pst*I and *Nhe*I, respectively. The HHRz with the mutated cleavage motif was generated by nested PCR and inserted in the 5' parental vector to replace the active HHRz domain using *Kpn*I and *Bbv*CI. Replacement of BDs in 3'ER vectors (p-3cBDopen or ss mutants) was done using the *Nhe*I/*Bbv*CI sites; BD replacement in 5'ER vectors (p-5cBDopen or ss mutants) was achieved using *Kpn*I/*Bbv*CI or *Kpn*I/*Bbs*I cleavage sites. Vector p-3BD(-) was obtained by replacing 3mBDopen with a short double-stranded oligonucleotide using the *Nhe*I and *Bbv*CI sites. Vectors p-5BD(-) and p-5BD(-)-ΔHH were generated by replacing 5mBDopen with an 8-mer oligonucleotide to form stem III of the HHRz followed by sub-cloning into the parental vector using *Kpn*I and *Bbv*CI. Vector p-5mBDopen-HH(-) was cloned using *Kpn*I and *Bbv*CI. Vectors for the expression of dual-targeting tsRNAs *trans*-splicing toward AFP and HCCA2 (p-3mBDopen-AFP+HCCA2 and p-3mBDopen-HCCA2+AFP) were generated by sub-cloning the dual BDs into the parental 3'ER vector using *Nhe*I and *Bbv*CI. Dual-targeting vectors p-3mBDopen-AFP+CD24 and p-3mBDopen-AFP+VEGF were generated by replacing the HCCA2 BD of p-3mBDopen-AFP+HCCA2 with the CD24 or VEGP BD using *Eco*RI and *Bbv*CI. Dual-targeting vectors p-3mBDopen-CD24+AFP and p-3mBDopen-VEGF+AFP were generated by replacing the HCCA2 BD p-3mBDopen-HCCA2+AFP with the CD24 or VEGF BD using *Eco*RI and *Nhe*I. To generate GFP expressing vectors for flow cytometry analyses, the *gfp* gene of pEGFP.C2 was PCR amplified and inserted into pGL3-Control to replace the firefly luciferase gene using *Hind*III and *Xba*I. The resulting GFP expression cassette comprising the SV40 promoter, *gfp*, SV40 polyA, and the SV40 enhancer was then sub-cloned into the self-generated MCS of the pVAX1-based *trans*-splicing vectors using *Bgl*II and *Sal*I. Sub-cloning of the GFP-expression cassette into pVAX1-AFP(E3-E6) was achieved using *Kpn*I and *Bam*HI. HPV-16 targeting 3'ER *trans*-splicing vectors p-3mBDE1a and p-3mBDE5 were generated by replacing the BD of the parental vector p-3mBDopen with BDs E1a and E5 using *Bam*HI and *Xho*I. Analogous, vectors p-3mBDE2 and p-3mBDE6 were generated by inserting BDs E2 and E6 using *Xho*I and *Xba*I. HPV-16-targeting 5'ER vector p-5mBDE1b was derived by replacing the AFP-targeting BD of p-5mBDopen with HPV-16 BD E1b using *Hind*III and *Bam*HI; vector p-5mBDE6-HH(-) was generated by replacing the AFP BD of p-5mBDopen-HH(-) with BD E6 using *Hind*III and *Bam*HI.

D, Supplemental References

1. Wang, Z.X., Wang, H.Y., and Wu, M.C. (2001). Identification and characterization of a novel human hepatocellular carcinoma-associated gene. British Journal of Cancer *85*, 1162-1167.
2. Huang, L.R., and Hsu, H.C. (1995). Cloning and expression of CD24 gene in human hepatocellular carcinoma: a potential early tumor marker gene correlates with p53 mutation and tumor differentiation. Cancer Research 55, 4717-4721.
3. Yang. X.R. *et al.* (2009). CD24 is a novel predictor for poor prognosis of hepatocellular carcinoma after surgery. Clinical Cancer Research: an Official Journal of the American Association for Cancer Research *15*, 5518-5527.
4. Zhao. Y.J., Ju, Q., and Li, G.C. (2013). Tumor markers for hepatocellular carcinoma. Molecular and Clinical Oncology *1*, 593-598.
